# The mitophagy targeted nano-assemblies with high Salvianolic acid B -loading sufficiently enhances osteoarthritis therapy

**DOI:** 10.1016/j.mtbio.2026.103601

**Published:** 2026-08-26

**Authors:** Yulin Jiang, Mingzhu Wang, Hanshuang Liang, Xuejie Hu, Liuwei Chu, Ranran Yang, Kang Wang, Ruhong Fang, Cailiang Shen, Xinming Wang, Wei Wei, Qingtong Wang

**Affiliations:** a Institute of Clinical Pharmacology, School of Pharmacy, Anhui Medical University, Key Laboratory of Anti-inflammatory and Immune Medicine, Ministry of Education, Anhui Provincial Engineering Technology Research Center for Anti-inflammatory and Immune Drugs, Hefei, 230032, China; b Department of Orthopedics and Spine Surgery, The First Affiliated Hospital of Anhui Medical University, Hefei, 230022, China; c Department of Immunology, School of Basic Medical Sciences, Center for Big Data and Population Health of IHM, Anhui Medical University, Hefei, 230032, China; d Department of Pharmacy, First Affiliated Hospital of Anhui Medical University, Hefei, 230022, China

**Keywords:** Osteoarthritis, Salvianolic acid B, Nano-assemblies, Mitophagy, Chondrocyte, PINK1

## Abstract

Osteoarthritis (OA) is characterized by chondrocyte degeneration and associated intracellular organelle dysfunction. Restoring impaired mitophagy represents a critical strategy for ameliorating OA-induced chondrocyte dysfunction and promoting cartilage repair, yet a significant therapeutic gap persists due to the lack of highly potent agents that can effectively restore mitophagy in OA chondrocyte. In this work, we identified Salvianolic acid B (SaB), a novel small-molecule agent, which binds to PINK1/Parkin proteins to stabilize them, promote their expression, and activate mitophagy. SaB nano-assemblies (SaB NAs) were fabricated via hydrogen bonding and π–π stacking between SaB and triphenylphosphine, achieving exceptional drug loading (74.4%) and mitochondrial-targeted synergistic therapeutic effects. *In vitro* and *in vivo* assessments confirmed that SaB NAs significantly outperformed free SaB in restoring mitochondrial function, facilitating chondrocyte recovery, prolonging therapeutic residence time and alleviating OA progression. The intra-articular delivery of SaB NAs represents a promising approach for treating OA and other joint inflammatory diseases.

## Graphical abstract

**Figure:**
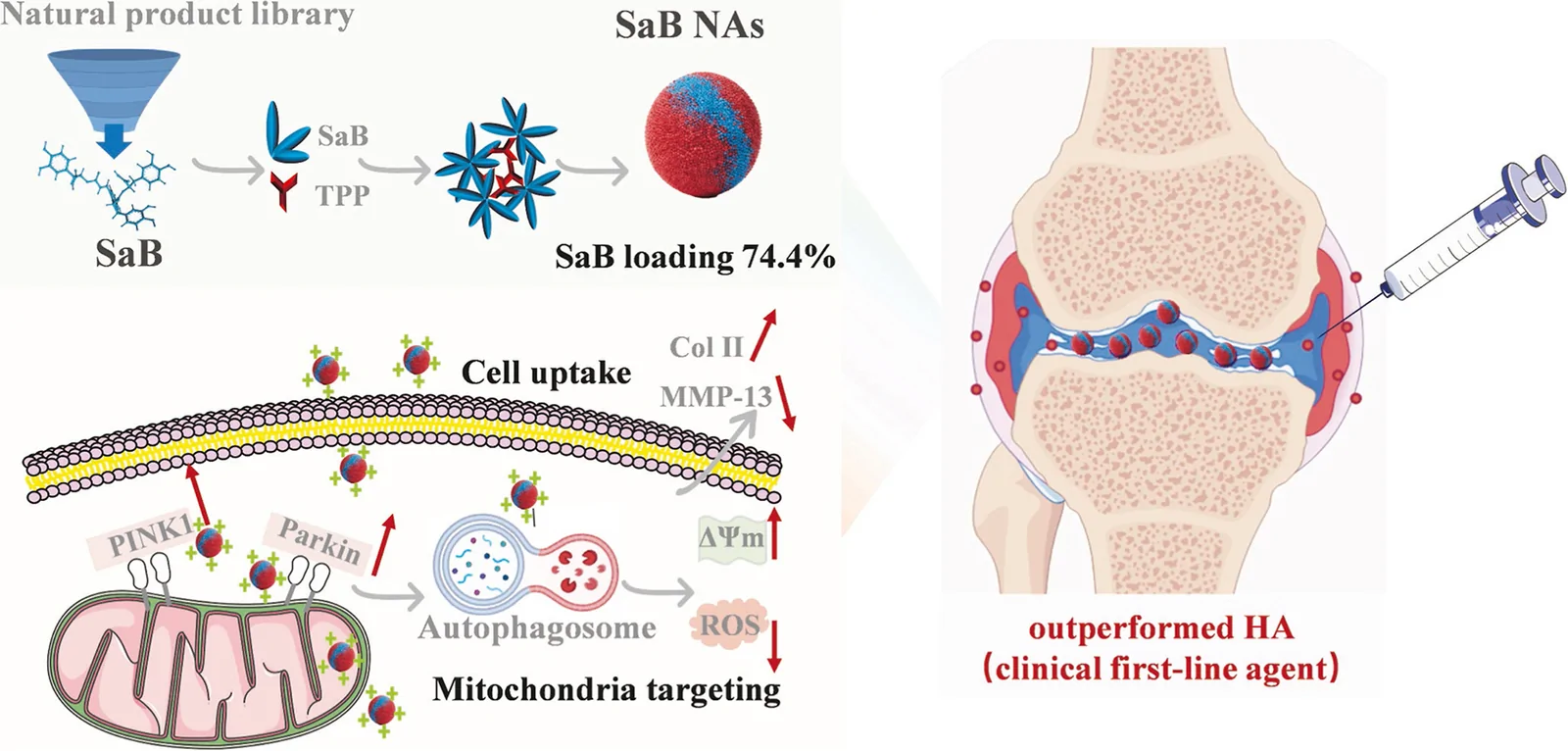

## 1 Introduction

Osteoarthritis (OA) is an age-related degenerative joint disease, affecting more than 650 million people worldwide [1]. Pathological progression of OA is characterized by chondrocyte damage and intracellular organelle dysfunction, accompanied by prominent structural and functional alterations of mitochondria within chondrocyte [2]. Mitophagy, a form of selective autophagy, eliminates damaged mitochondria to maintain cartilage matrix homeostasis [3]. Recently, accumulating evidence suggests that impaired mitophagy is tightly involved in the progression of OA [[4], [5], [6]]. Mitophagy impairment induces persistent accumulation of dysfunctional mitochondria, which triggers chondrocytes apoptosis and extracellular matrix degradation, ultimately driving cartilage degeneration. Consequently, activating impaired mitophagy represents a promising therapeutic strategy to ameliorate OA chondrocyte dysfunction and enhance cartilage repair and regeneration. Despite active research on small-molecule mitophagy modulators, none have yet advanced to clinical applications [7]. While clinical small-molecule drugs like curcumin and metformin may initiate recognition of impaired mitophagy, their therapeutic efficacy remains limited [8], highlighting the critical need for highly bioactive drug formulations to ensure effective clinical application. The PINK1/Parkin pathway, a canonical ubiquitin-dependent mitophagy pathway, mediates the ubiquitination of mitochondrial outer membrane components and then subjected to bind LC3B and initiate autophagosome formation [9]. Activating impaired mitophagy by PINK1/Parkin axis represents a promising strategy for precision molecular therapy in OA [10].

Salvianolic acid B (SaB), a natural polyphenolic compound, has been well documented to exert anti-apoptotic, antioxidant and anti-inflammatory activities, alongside mitochondrial protective effects in multiple disease contexts [11].SaB has been developed for the treatment of various disorders. Nevertheless, whether SaB modulates the PINK1/Parkin mitophagy pathway in chondrocytes remains poorly defined. Using molecular docking combined with cellular functional assays, we identified SaB as a candidate regulator of the PINK1/Parkin pathway, which may serve to restore mitophagy in chondrocytes. Intra-articular injection is the major administration strategy for OA therapeutics. However, drug efficacy is frequently compromised by insufficient local accumulation and short retention time of therapeutic agents within the joint cavity [12]. The nanodrug formulations have emerged as potent solution to overcome the limitations in conventional drug molecules [13]. Notably, pure drug nano-assemblies (PDNAs) formed through the self or co-assembly of pure drug molecules, have attracted considerable attention [[14], [15], [16], [17]]. Functioning as both carriers and therapeutic agents, carrier-free PDNAs exhibit an ultra-high drug loading capacity. Furthermore, co-assembly behaviour enables the simultaneous delivery of multi-drug combinations for synergistic therapeutic strategies [18]. For polyphenols, existing PDNAs techniques such as polymer coupling and metal coordination require complex syntheses and bring potential biological toxicity [[19], [20], [21]]. The development of a novel SaB-based PDNA via a green and efficient synthesis strategy is critical for effectively modulating mitophagy and alleviating OA-associated chondrocyte dysfunction.

**Scheme 1:**
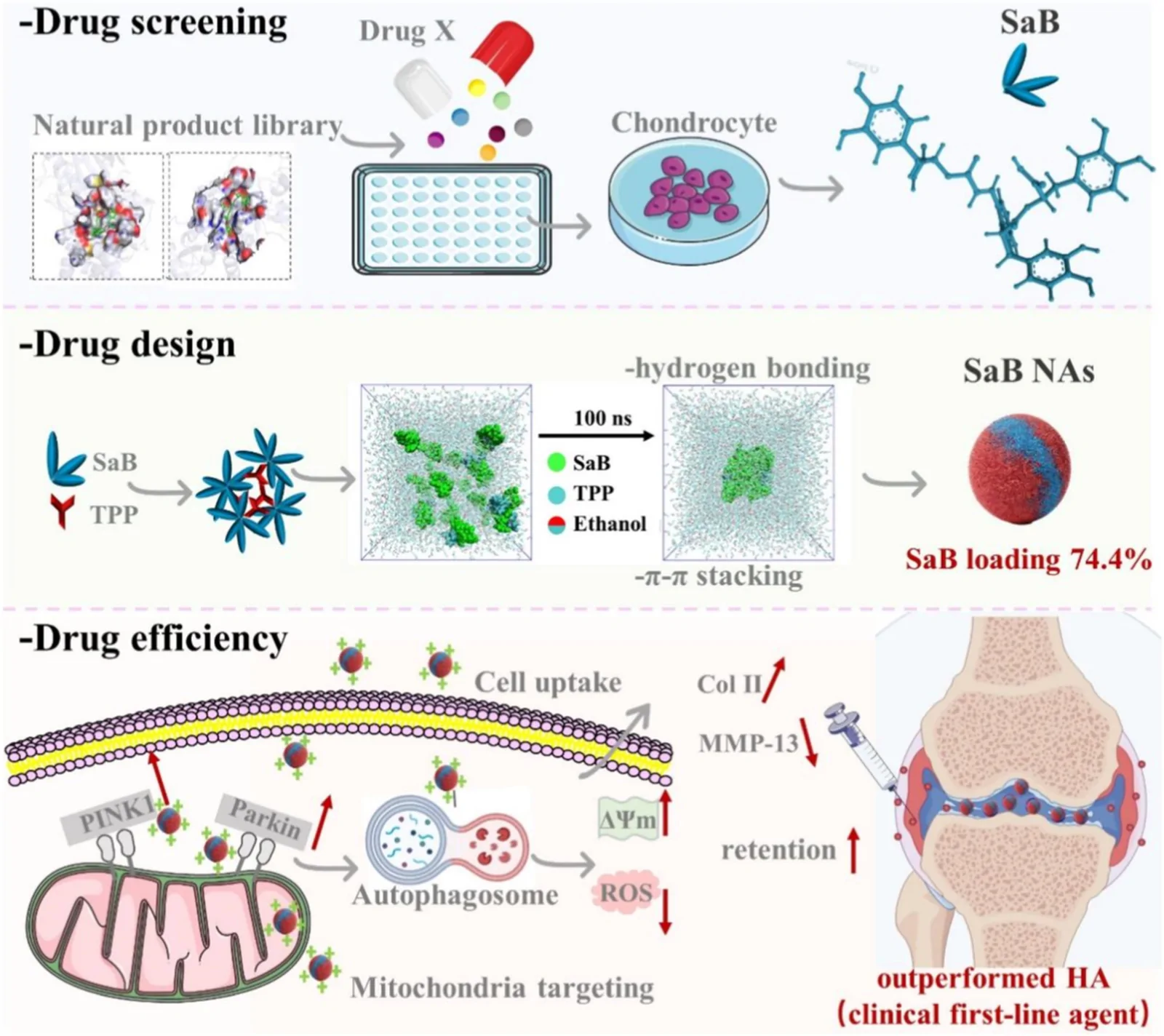
Self-assembly nanodrug for SaB: from drug screening, drug design to drug efficiency.

Structurally, SaB features a rigid aromatic backbone decorated with multiple hydroxyl moieties, enabling extensive hydrogen-bonding networks and π-π stacking interactions. This design enables the formation of stable supramolecular architectures [22]. Triphenylphosphine (TPP) is a lipophilic cation that can achieve mitochondrial targeting due to electrostatic interactions with the negative mitochondrial membrane potential (ΔΨ≈−150 to−180 mV) [23]. This study aimed to develop a rapid and convenient method for synthesizing SaB-based nano-assemblies (SaB NAs) through self-assembly between SaB and TPP. Preliminary data showed that SaB-NDs, formed via the hydrogen bonding and π-π interactions between SaB and TPP, can target mitochondria and activate impaired mitophagy through the PINK1/Parkin pathway, thereby restoring chondrocyte function. Furthermore, compared with free SaB monomers, SaB NAs exhibits enhanced cellular uptake efficiency and prolonged drug retention. This method demonstrates a green and facile strategy for synthesizing highly bioactive SaB NAs, with promising therapeutic potential for OA treatment (Scheme 1).

## 2 Materials and methods

### 2.1 Materials

SaB (≥98% purity) was purchased from Aladdin Biochemical Technology Co., Ltd. (China). TPP (≥99% purity), methanol, ethanol, acetone and dichloromethane (DCM) were obtained from Macklin Biochemical Technology Co., Ltd. (China). Cyanine5 amine (Cy5-NH_2_) and DPPH were purchased from Solarbio Science & Technology Co., Ltd. (China). Sodium hyaluronic injection (HA), used as a positive control, was obtained from Seikagaku Corporation. IL-1β (Cat. No. 211-11B-10UG) and BCA Protein Assay Kit were obtained from Thermo Fisher Scientific, Inc. (USA). Primary and secondary antibody diluents, RIPA Lysis buffer, PMSF, Phosphatase inhibitor cocktail were obtained from Beyotime Biotech, Inc. China). MMP-13 (Cat. No. 18165-1-AP), Collange II (Cat. No. 28459-1-AP), PINK1 (Cat. No. 23274-1-AP), Parkin (Cat. No. 14060-1-AP) and LC3B (Cat. No. 18725-1-AP) were bought from Proteintech Group, Inc. (China). Antibodies against glyceraldehyde-3-phosphate dehydrogenase (GAPDH, Cat. No. GB15004-100) and TRIzol reagent were purchased from Servicebio Technology Co., Ltd. (China). HRP-conjugated secondary antibodies were obtained from Jackson ImmunoResearch Inc. (USA). SDS-PAGE Loading Buffer, SuperSignal ECL Chemiluminescent Substrate Kit and Cell Counting Kit-8 (CCK-8) were purchased from Labgic Technology Co., Ltd. (China). Dulbecco's modified Eagle medium/nutrient mixture F-12 medium (DMEM/F12) and fetal bovine serum (FBS) were purchased from Wisent Biotechnology (Nanjing) Co., Ltd. (China). Trypsin-EDTA solution, Penicillin-Streptomycin, ROS Assay Kit (Cat. No. S0035S), ATP Assay Kit (Cat. No. S0033M), JC-1 Mitochondrial Membrane Potential Assay Kit (Cat. No. C2006), Mito-Tracker Green (Cat. No. C1048), Hoechst 33342 (Cat. No. C1027) were obtained from Beyotime Biotech, Inc. (China). Immunochemistry Kit PV9000 and DAB Substrate Kit were purchased from Zhongshan Golden Bridge Biotechnology Co., Ltd. (China). Deionized (DI) water was produced by a water filtration system (Merck Milli-Q, USA).

### 2.2 Preparation of SaB NAs

To prepare SaB NAs, Solution A (1 mM) was prepared by dissolving SaB in 2 mL deionized water, while Solution B was prepared by dissolving TPP in 2 mL ethanol. Solution B was then incrementally added to Solution An under magnetic stirring for 10, 20, 40, and 120 min, respectively, with corresponding molar ratios of Solution A to Solution B set at 1:1, 2:1, 3:1, and 4:1. For comparative analysis, the ethanol in Solution B was systematically replaced with deionized water, methanol, acetone, and DCM. The resulting suspensions were ultrafiltered using 100 kDa molecular-weight-cut-off (MWCO) polysulfone membranes to remove unreacted SaB and TPP. The samples were redispersed in deionized water or freeze-drying for subsequent characterization.

### 2.3 Characterization

To investigate the morphological features and elemental composition of the samples, scanning electron microscopy (SEM, SU8600, HITACHI, Japan) coupled with energy-dispersive X-ray spectroscopy (EDS) and transmission electron microscopy (TEM, Talos F200, Thermo Fisher, USA) were employed. The hydrodynamic diameter and zeta potential of the nanoparticles were quantified using Mastersizer3000 (Malvern, UK). For chemical characterization, Fourier-transform infrared spectroscopy (FTIR) was conducted on a Nicolet IS5 spectrometer (Thermo Fisher, USA) within the spectral range of 4000-500 cm^-1^, while ultraviolet-visible absorption (UV) spectra were recorded with a UV-1900i spectrophotometer (SHIMADZU, Japan). To elucidate the molecular interactions and structural bonding modes, proton nuclear magnetic resonance (^1^H NMR) analysis was carried out on a 400 MHz AVANCE III spectrometer (Bruker, Germany).

### 2.4 HPLC-MS

High-performance liquid chromatography (HPLC, 1260 Infinity Ⅱ, Agilent, USA) was conducted to determine the concentration of SaB at a wavelength of 288 nm. Chromatographic separation was performed on an Eclipse XDB-C18 column (4.6 mm × 150 mm, 5 μm) at 30°C. The mobile phase was acetonitrile with a flow rate of 1.5 mL/min.

### 2.5 In vitro release assay of SaB NAs

The drug content of Sal B was determined using UV-Vis spectrophotometry with its characteristic absorption peak at 287 nm as the detection wavelength. First, precisely weigh 5 mg of Sal B reference substance, dissolve it in acetonitrile, and dilute to a specified volume in a volumetric flask. Then, prepare a series of concentration gradient solutions (0, 10, 20, 30, 40, 50 μg/mL) and measure their absorbance at 287 nm. Next, conduct linear regression analysis using drug concentration as the abscissa and absorbance as the ordinate to plot the standard curve and calculate the regression equation. Subsequently, precisely weigh 5 mg of Sal B-loaded nanoparticles (NAs), dissolve them in acetonitrile, and prepare a 35 μg/mL solution. After filtration through a 0.22 μm membrane, collect the filtrate and measure its absorbance at 287 nm. Finally, substitute the measured absorbance into the standard curve to calculate the drug loading rate and drug release rate of the sample.

### 2.6 DPPH assays

DPPH free radical scavenging test was performed according to the method described in a previous study [24]. DPPH stock solutions (0.1 mM in ethanol) were prepared and stored in dark to maintain stability. Subsequently, SaB NAs suspensions were introduced into 10 mL of DPPH solution, resulting in final SaB NAs concentrations of 2, 4, 8, 20 and 30 μg/mL. The reaction mixtures were incubated in the dark for a specified duration, after which absorbance measurements were conducted at 200-800 nm using UV–vis spectroscopy. Scavenging efficiency was quantified via the formula: S = [1 – (A*m* – A*n*)/A_*0*_] ×100%, where A*m* is the absorbance of the mixture of DPPH and SaB NAs resuspension, A*n* is the absorbance of SaB NAs in ethanol solution, and A_*0*_ is the absorbance of DPPH solution without SaB NAs.

### 2.7 Molecular dynamics simulation

A cubic simulation box (6 nm × 6 nm × 6 nm) was initialized, containing 30 SaB and 15 TTP molecules arranged randomly to form the initial system. Molecular dynamics (MD) simulations were conducted using GROMACS 2022.2 [25] under isothermal-isobaric (NPT) ensemble conditions with periodic boundary constraints. The system was parameterized using the General Amber Force Field (GAFF), with hydrogen bond constraints applied via the LINCS algorithm at a 2 fs time step. Electrostatic interactions were calculated using the Particle-Mesh Ewald (PME) method, while non-bonded interactions were truncated at 10 Å with a neighbor list update frequency of 10 steps. Temperature control was maintained at 298.15 K using the V-rescale thermostat, and pressure regulation at 1 bar was achieved through the Parrinello-Rahman barostat. The simulation protocol included three stages: Firstly, initial energy minimization was performed via the steepest descent algorithm to resolve atomic clashes. Then, a 100 ps NVT equilibrium simulation was conducted at 298.15 K. Finally, two distinct systems were simulated for 100 ns each, with structural snapshots saved every 10 ps. The visualization of the simulation results was accomplished using the Gromacs embedded program and VMD.

### 2.8 Molecular docking

Salvianolic acid B structure model was downloaded from PubChem. Parkin (PDB ID:5C1Z) and PINK1(PDB ID:9EII) structure model was downloaded from RCSB. The protein was processed on the Maestro 11.9 platform, excluding all water molecules and adding polar hydrogen atoms. Molecular docking calculations were performed by the Glide module within the Schrödinger Maestro software. Docking was executed in Standard Precision mode with default Glide parameters.

### 2.9 Cell extraction and culture

Articular cartilage was extracted from the knee joints of 4-week-old rats. The tissue was minced into 1.0 mm^3^ fragments and digested with 0.2% type II collagenase in DMEM for 12 h at 37°C. Chondrocytes were then isolated and cultured in DMEM/F12 supplemented with 10% fetal bovine serum at 37°C under 5% CO_2_.

### 2.10 Cell viability

For the CCK-8 assay, chondrocytes were seeded in 96-well plates (1 × 10^4^ cells/well) and cultured in a humidified incubator at 37°C with 5% CO_2_. The culture medium was subsequently replaced with fresh medium containing SaB or SaB NAs. The viability and proliferation of chondrocytes co-cultured with SaB or SaB NAs were assessed on day 1, 3, 5 and 7 using a Live/Dead kit and CCK-8, respectively. The cells were observed by under a cell discoverer 7 high-throughput live-cell workstation (Zeiss, Germany). The optical density (OD) were detected at 450 nm by microplate reader (Tecan, Switzerland).

### 2.11 Intracellular uptake

Chondrocytes were seeded in glass-bottom cell culture dishes (2x10^5^ cells/well). Cy5-labeled SaB and SaB NAs were co-cultured with chondrocytes for 1 and 4 h. Then, the chondrocytes were treated with Hoechst 33342 (diluted 1:1000) for 20 min. Subsequently, Zeiss Cell Discoverer 7 microscope was employed to observe and analyze the chondrocytes.

### 2.12 Mitochondrial targeting

Cy5-labeled SaB and SaB NAs were co-cultured with chondrocytes for 1 and 4 h. Mitochondria were stained with Mito-Tracker Green (incubated at 37°C for 30 min), and cell nuclei were counterstained with Hoechst 33342 (diluted 1:1000, incubated for 20 min). After three washes with PBS, cells were imaged using a Zeiss Cell Discoverer 7 microscope. Pearson correlation analysis, fluorescence intensity and line-scan intensity profiles for co-localization were quantified using ImageJ.

### 2.13 Investigation of intracellular ROS, ATP, mitochondrial membrane potential, and mitochondrial morphology

Chondrocytes were seeded in 6-well plates (5x10^5^ cells/well), and IL-1β was added to treat cell. The group of SaB and SaB NAs were supplemented and cultured for 12 h. For ROS detection, the cells were treated with 1 ml diluent DCFH-DA for 20 min. After three washes with PBS, cells were observed with a Zeiss Cell Discoverer 7 microscope. For ATP measure, the cells were collected and lysed in RIPA buffer. The supernatant was isolated by centrifugation and was added to 100 μL ATP detection working solution in a 6-well plate, and the luminescence was measured as relative light units (RLU) results using a SuPerMax 3100 microplate reader (Tecan, Switzerland). For mitochondrial membrane potential measurement, the cells were stained with the JC-1 Membrane Potential Detection Kit for 20 min, washed 3 times, and then observed under the same microscope. For mitochondrial morphology observation, the cells were scraped with a cell scraper, centrifuged for 5 min, then 2% glutaraldehyde fixative solution was slowly added and the cells were fixed at 4°C for 5 h. After washing with PBS, the samples were fixed with 1% tetracarbonyl osmium for 2 h. Subsequently, the samples were dehydrated, embedded with fresh resin, sectioned, stained, and finally observed under a transmission electron microscope (TEM, Talos L120C G2, Thermo Fisher, USA).

### 2.14 qRT-PCR

Total RNA from chondrocytes cells were isolated using TRIzol Kit (Servicebio, China) following the manufacturer's protocols. RNA was reversely transcribed with SYBR Green (Takara, China) according to manufacturer's instructions. PCR amplification conditions were as follows: 40 cycles at 95°C for 5 min, 95°C for 10 s, 60°C for 20 s, and 72°C for 20 s. Primer sequences were provided in Supplementary Table S1. Fold changes of targeted mRNAs were calculated using the 2− ΔΔCt method, with GAPDH serving as the reference gene.

### 2.15 Western blots

Proteins were obtained from chondrocytes by RIPA lysis and extraction buffer. The protein concentrations were determined using a BCA Protein Assay Reagent Kit following the manufacturer's protocol. Protein samples were mixed with loading buffer, denatured at 95°C for 10 min, and separated by 10% SDS-PAGE. Proteins were transferred to PVDF membranes and blocked with 5% skim milk for 2 h at 4°C. After blocking, membranes were incubated with primary antibody overnight at 4°C, followed by secondary antibody incubation at room temperature for 2 h. Finally, the protein content was visualized using a Tancon 5200 automatic chemiluminescence image analysis system. The gray values were quantified (normalized to GAPDH) with ImageJ software.

### 2.16 Sequencing

An in vitro OA model was established using primary human chondrocytes stimulated with IL-1β (10 ng/mL, 24 h). Total RNA was extracted using TRIzol® reagent (Invitrogen) following the manufacturer's protocol. High-throughput RNA sequencing was performed on the Illumina NovaSeq platform in paired-end mode. Differential gene expression analysis was conducted using DESeq2. Genes were defined as differentially expressed (DEGs) when meeting |log_2_ fold change| ≥ 1 and adjusted p-value (FDR) ≤ 0.05. Gene Ontology (GO) enrichment and Kyoto Encyclopedia of Genes and Genomes (KEGG) pathway analysis were performed using cluster Profiler (v4.2.4) with p < 0.05 as the significance threshold.

### 2.17 Cellular thermal shift assay (CETSA)

Cells were seeded in 6-well plates at a density of 2.0 × 10^5^ cells per well and cultured overnight to allow attachment. The medium was then replaced with fresh medium containing either SaB (100 μM) or an equivalent volume of PBS as a vehicle control, and cells were incubated for 2 h at 37°C with 5% CO_2_. After treatment, cells were harvested by trypsinization, washed twice with ice-cold PBS, and resuspended in PBS supplemented with a protease inhibitor cocktail. Cell suspensions were divided into aliquots and heated individually at the indicated temperatures (37, 42, 47, 52, 57, 62, and 67°C) for 3 min using a thermal cycler (Long Gene, T20), followed by immediate cooling at room temperature for 3 min. The heated samples were then subjected to three freeze-thaw cycles to ensure complete cell lysis. Cellular debris was removed by centrifugation at 4°C. The supernatants were collected, and protein concentrations were determined using the BCA protein assay kit. Equal amounts of protein (30 μg per lane) were separated by 10% SDS-PAGE and transferred onto PVDF membranes. The membranes were blocked with 5% non-fat milk in TBST for 2 h at room temperature and then incubated overnight at 4°C with primary antibodies against PINK1 (1:1000) and Parkin (1:1000). After washing with TBST, the membranes were incubated with HRP-conjugated secondary antibodies (1:10000) for 2 h at room temperature. Immunoreactive bands were visualized using an enhanced chemiluminescence (ECL) substrate and captured using an ImageQuant LAS 500 imager (GE Healthcare Systems, Chicago, IL, USA).

### 2.18 Cycloheximide (CHX) chase assay

Cells were plated in 6-well plates at 2.0 × 10^5^ cells per well and allowed to adhere overnight. Cells were then pre-treated with SaB (100 μM) or PBS for 24 h to ensure adequate drug-protein engagement prior to protein synthesis inhibition. Subsequently, 10 μg/mL of CHX (MedChemExpress, HY-12320) was added to block *de novo* protein synthesis, and cells were further incubated for the indicated time periods (0, 3, 6, 9, and 12 h). At each time point, cells were harvested, washed with ice-cold PBS, and lysed in RIPA buffer (Beyotime, P0013C) supplemented with 1× protease inhibitor cocktail and 1 mM PMSF on ice for 30 min. Lysates were clarified by centrifugation at 4°C, and protein concentrations were determined by the BCA assay. Equal amounts of protein (30 μg) were resolved by SDS-PAGE and analyzed by western blotting as described above, using antibodies against PINK1, Parkin, and GAPDH.

### 2.19 siRNA transfection

Chondrocytes were seeded in 6-well plates at a density of 2.0 × 10^5^ cells per well in complete DMEM/F-12 medium supplemented with 10% FBS and 1% penicillin-streptomycin. After 24 h of incubation to allow cell attachment and reach 60-70% confluence, the culture medium was replaced with antibiotic-free medium. Cells were transiently transfected with siRNA-PINK1 #2 (50 nM, sense: 5′-GUCAGGAGAUCCA GGCAAUUUTT-3′, antisense: 5′-AAAUUGCCUGGAUCUCCUGATT-3′; GENERAL BIOL, Chuzhou, China) or a scrambled negative control siRNA (NC siRNA, 50 nM; GENERAL BIOL) using NanoTrans™ Transfection Reagent Plus (CYTOCH, CT0005) according to the manufacturer's protocol. Briefly, siRNA and NanoTransTM Transfection Reagent Plus were each diluted in Opti-MEM reduced-serum medium (Gibco, 31985-070) and incubated at room temperature for 5 min. The two dilutions were then combined, mixed gently, and incubated for an additional 10 min at room temperature to allow complex formation. The siRNA-lipid complexes were added dropwise to the cells, and the plates were gently rocked to ensure even distribution. After 6 h of transfection, the medium was replaced with complete culture medium. At 24 h post-transfection, cells were subjected to subsequent treatments as indicated.

### 2.20 Rat OA model and drugs injection

The animal experiment was approved by the Ethics Committee of Anhui Medical University (PZ-2024-049). OA was induced via destabilization of the medial meniscus combined with anterior cruciate ligament transection (DMM + ACLT) as previously described [26]. The male Sprague Dawley (SD) rats (280 ± 20 g, 8 weeks old, n = 48) were selected for animal experiments. After the rats were fasted and anesthetized, the medial meniscus was partially excised, and the anterior cruciate ligament (ACL) was transected. ACL integrity was confirmed via the anterior drawer test. Following wound closure, the incision was sutured in a layered fashion. The sham-operated control group (n = 8) underwent anesthesia and skin incision only, with no ligament or meniscal manipulation. The OA rats (n = 40) were divided into four groups, and 100 μL of PBS (n = 8), SaB (n = 8), TPP (n = 8), SaB NAs (n = 8) and HA (a positive control drug) (n = 8) were respectively injected into the knee joint at the second and fifth weeks after surgery.

### 2.21 Biodistribution and retention *in vivo*

Cy5-labeled SaB and Cy5-labeled SaB NAs were separately injected into the knee joints of rats. Fluorescence signals were quantified at 1, 3, 5, 7, 14 and 28 days post-administration using the IVIS Spectrum® *in vivo* imaging system (PerkinElmer, Waltham, MA, USA). Region-of-interest (ROI) analysis was performed on all images using ImageJ, with fluorescence intensity values (mean ± SD) extracted from the knee joint area.

### 2.22 Gait analysis

Before euthanasia at 5 weeks, the rats were subjected to gait experiments. Rats were individually placed in the gait analysis corridor for habituation prior to data acquisition. Automated gait recording was conducted during free ambulation using the CatWalk XT system (XR-FP101, China), capturing full gait cycles. Key parameters were extracted via CatWalk software.

### 2.23 Micro computed tomography (CT)

Micro-CT imaging of rat knee joints was performed using the Quantum GX Micro CT Imaging System (VNC-102, China) with the following parameters: 45 mm fField of view, 86 mm axial scan range and 4 s/bed scan speed. Then, three-dimensional (3D) images were reconstructed using multimodal 3D visualization software. Region of interest (ROI) analysis was performed on the subchondral bone compartment using Avatar software.

### 2.24 Histological staining

At the 5th week post-operation, all rats were euthanized. The knee joint specimens were collected, fixed in 4% paraformaldehyde, decalcified, and then embedded for sectioning. After deparaffinization, hematoxylin-eosin (H&E), Safranin O-Fast Green (SO&FG), and toluidine blue (TB) staining were performed with standardized durations. The histopathological analysis was conducted via modified Mankin score and Osteoarthritis Research Society International (OARSI) cartilage injury score [27]. Furthermore, the major organs including the heart, liver, spleen, lung, and kidney from rats were collected. Tissues were fixed in 4% paraformaldehyde, embedded in paraffin, sectioned, and stained with H&E.

### 2.25 Immunofluorescence staining

Tissue sections were subjected to xylene-mediated deparaffinization (10 min each in xylene I and II), followed by rehydration through a graded ethanol series down to distilled water. Sections were permeabilized with 0.05% Triton X-100 for 20 min. For antigen retrieval by the trypsin-based method, 0.1% trypsin digestion solution was drop-wise applied onto the sections, which were then incubated in a 37°C oven for 30 min to expose antigenic epitopes. Non-specific binding was blocked with 5% BSA at room temperature for 1 h. Primary antibodies against LC3B (1:200) and TOM20 (1:500) were applied onto the tissue surface, and incubation was performed overnight at 4°C. On the following day, after the sections had been equilibrated at room temperature for 30 min, they were incubated with fluorophore-conjugated secondary antibodies for 2 h under light-protected conditions. Cell nuclei were counterstained with DAPI (Biosharp, Cat. No. BL105A). Finally, images were acquired using a Leica SP8 laser-scanning confocal microscope.

### 2.26 Immunohistochemical staining

The knee joint sections were blocked with 5% bovine serum albumin (BSA) at room temperature for 30 min to prevent nonspecific protein binding, followed by incubation overnight at 4°C with primary antibodies. Sections were then incubated with horseradish peroxidase-conjugated secondary antibodies for 1 h at room temperature, and positive staining was visualized using a DAB detection system. After chromogen development, sections were counterstained with hematoxylin, dehydrated through a graded ethanol series, cleared in xylene, mounted with mounting medium, and digitally scanned using a PANNORAMIC MIDI II slide scanner for quantitative analysis.

### 2.27 Statistical analysis

Data were statistically analyzed using GraphPad Prism 9 software. One-way analysis of variance (ANOVA) was used for comparisons among multiple groups, Two-way ANOVA was applied to analyze group differences for the two factors, and *t*-test was applied to determine differences between two groups. A *P*-value < 0.05 was considered statistically significant.

## 3 Results and discussion

### 3.1 Activation of PINK1/parkin-mediated mitophagy by SaB

**Fig. 1:**
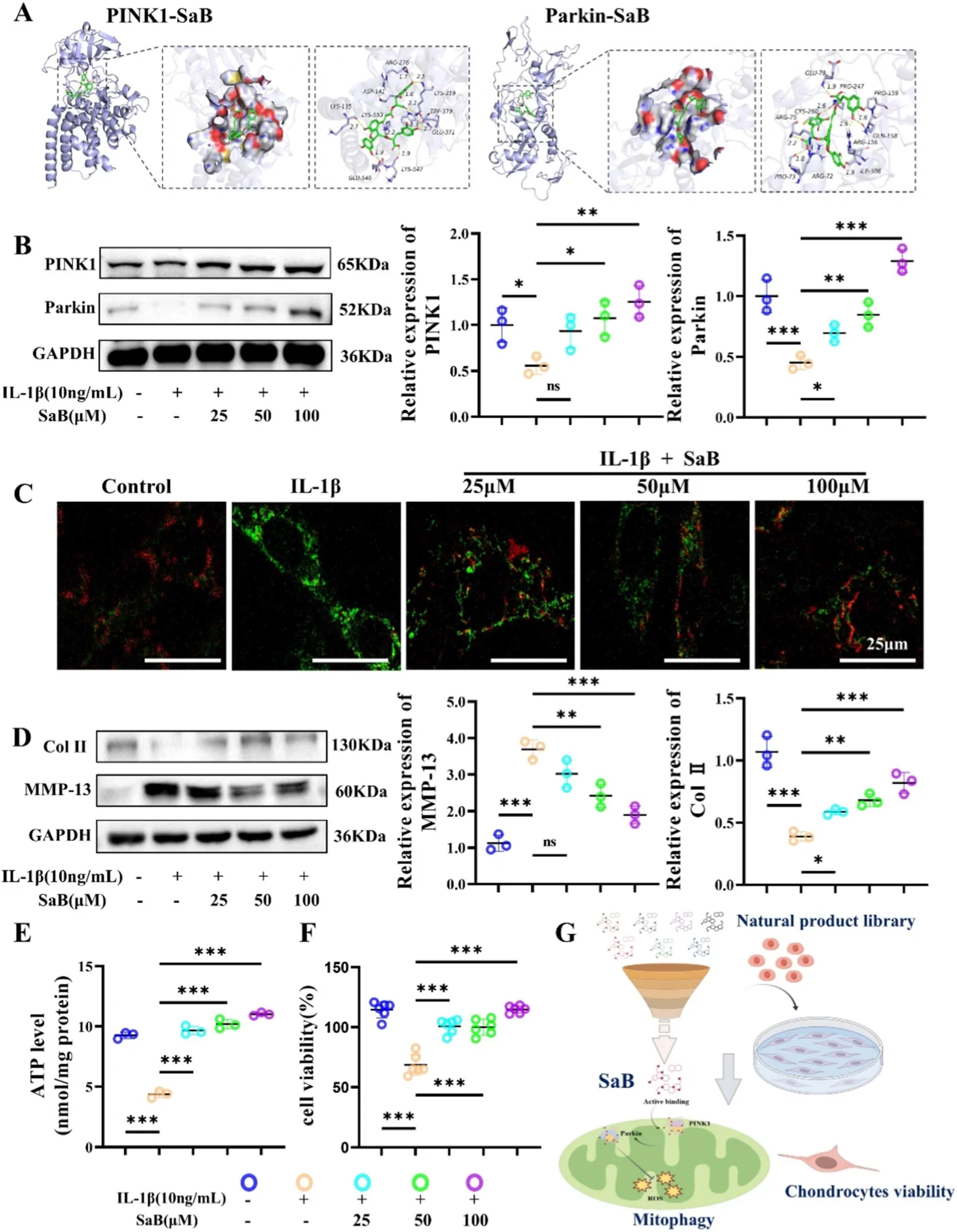
Activation of PINK1/Parkin by SaB protected cartilage ECM anabolic/catabolic metabolism. A. Molecular docking of SaB-Pink1 and SaB-Parkin complexes. B. The protein expression levels of PINK1, Parkin in IL-1β-stimulated chondrocytes treated with SaB (0, 25, 50, or 100 μM) and quantification analysis. C. Representative JC-1 fluorescence images showing mitochondrial membrane depolarization in IL-1β-stimulated chondrocytes treated with SaB (0, 25, 50, or 100 μM) (scale bar, 25 μm). D. The protein expression levels of Col II, MMP-13 in IL-1β-stimulated chondrocytes treated with SaB (0, 25, 50, or 100 μM) and quantification analysis. E. Intracellular ATP level and F. Chondrocyte viability in IL-1β-stimulated chondrocytes treated with SaB (0, 25, 50, or 100 μM). G. Schematic illustrating SaB screening and therapy. The values are presented as the means ± SDs. *p < 0.05, **p < 0.01, ***p < 0.001, ns, not significant. One-way ANOVA was used for anaysis.

The PINK1-Parkin axis initiates autophagosome formation, demonstrating significant therapeutic potential for OA [6]. Through high-throughput screening of a natural product library, SaB was identified as a small-molecule agent that binds to PINK1 and Parkin, thereby stabilizing these proteins and promoting their expression. By molecular docking, a possible binding between SaB and the PINK1 protein at the ARG276, LYS319, GLU371, LYS547, GLU546, LYS135, ASP141 and TRP379 sites, with a binding energy of -10.152 kcal mol^-1^. Furthermore, SaB and the Parkin bind at residues GLU79, GLN158, ARG156, ARG72, ARG75, CYS260, ILE306, PRO159, PRO247 and PRO73, with a binding energy of -14.090 kcal mol^-1^ (Fig. 1A). The SaB was identified as a potential modulator of the PINK1/Parkin pathway, which was expected to ameliorate OA chondrocyte dysfunction. The chondrocytes were treated with different concentrations of SaB (0, 25, 50, 100, 150 or 200 μM) for 1, 3, 5 or 7 days. CCK-8 assay results confirmed non-toxicity at 0-100 μM (Fig. S1), and this concentration range was selected for subsequent experiments. Western blot experiments were performed as shown in Fig. 1B. Following IL-1β treatment to simulate the OA microenvironment, the expression of PINK1 and Parkin was significantly reduced. After co-treatment with SaB (25, 50, or 100 μM), the expression of PINK1 and Parkin was enhanced in a dose-dependent manner. The mitochondrial membrane potential (MMP) was detected using JC-1, a dual-emission fluorescent probe, with monomers (green) at low MMP and aggregates (red) at high MMP. IL-1β treatment significantly reduced MMP in chondrocytes, while SaB restored MMP, evidenced by increased red fluorescence intensity and decreased green fluorescence, with the effect intensifying at higher SaB concentrations (Fig. 1C and Fig. S2). Consequently, compared with the IL-1β group, SaB treatment significantly promoted the production of the cartilage extracellular matrix (ECM) components (collagen type II, Col II), and suppressed the expression of collagen-degrading enzyme (matrix metalloproteinase-13, MMP-13) (Fig. 1D). Additionally, intracellular ROS levels detection revealed that SaB effectively reduced cellular ROS (Fig. S3). This reduction in oxidative stress contributes to a more favorable microenvironment for cartilage repair. These results demonstrate that SaB effectively rescues IL-1β-induced mitochondrial ATP reduction (Fig. 1E) and cell inactivation (Fig. 1F) in chondrocytes. As illustrated in Fig. 1G, SaB was identified as a novel activator of the PINK1/Parkin mitophagy pathway and significantly ameliorated mitochondrial dysfunction and chondrocyte degeneration in OA.

### 3.2 Preparation and characterization of SaB NAs

**Fig. 2:**
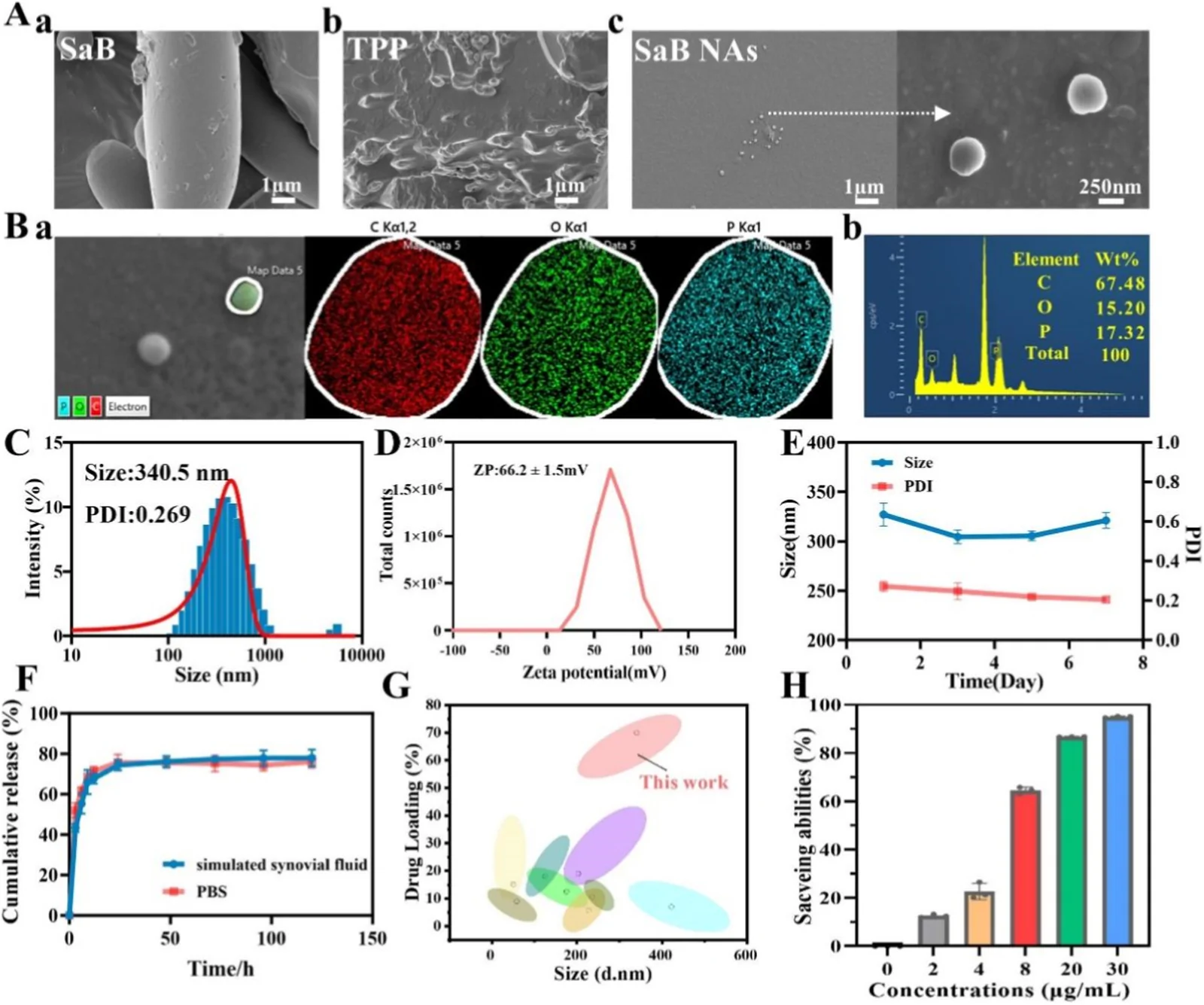
Physicochemical properties of SaB NAs. A. SEM images of a) SaB, b) TPP and c) SaB NAs powder. B. a) EDS mapping and b) patterns on SaB NAs corresponding to the SEM. C. Size distribution of SaB NAs. D. Zeta potential of SaB NAs solution, the inset shows the charge of SaB and TPP. E. DLS results regarding the size and PDI of SaB NAs for seven days. F. Drug release of SaB from SaB NAs in PBS and simulated synovial fluid. G. Drug loading capacity of SaB NAs vs. other polyphenol-based nanocarriers [21,[30], [31], [32], [33], [34], [35], [36]]. H. Radical scavenging ability variation with SaB NAs concentration.

To enhance the efficacy of this free drug, the SaB nano-assemblies (NAs) were developed by a simple process. The SEM of SaB, TPP and SaB NAs powders was illustrated in Fig. 2A. SAB NAs with a nanosphere morphology were formed through self-assembly from irregular products SaB and TPP in solutions. The TEM image of SaB in Fig. S4 demonstrates that the nanoparticles with higher density are uniformly dispersed. The EDS mapping in Fig. 2Ba demonstrated that the C, O, and P have a uniformly distribution on the surface of SaB NAs. The introduction of P, a characteristic element of TPP, indicates successful self-assembly of SaB NAs. The high relative amount of P element in Fig. 2Bb further confirms the uniform self-assembly through SaB and TPP. From the DLS analysis in Fig. 2C, the average particle size of SaB NAs was 340 nm, and the polydispersity index (PDI = Mw/Mn) was 0.268. As reported, nanoparticles exceeding 250 nm in size delay rapid clearance from the joint cavity through blood capillaries [28,29]. By measuring, the surface charge of SaB NAs solution was 66.2 ± 1.5 mV (Fig. 2D). Due to the carboxyl group, SaB exhibits a negative charge in solution. Upon addition of TPP, a lipophilic cation, SaB NAs acquire a positive charge. Positively charged nanoparticles exhibit enhanced affinity for the negatively charged cartilage matrix, facilitating targeted delivery [12]. The particle size and PDI did not change significantly over a duration of seven days (Fig. 2E), indicating the good stability of SaB NAs. And in culture medium, serum-containing medium, and simulated synovial fluid, the SaB NAs also maintained stability over a duration of seven days (Fig. S5). As shown in Fig. S6, HPLC analysis of SaB NAs revealed characteristic retention times of 10 min and 21 min, corresponding to free SaB and TPP, respectively. Drug release studies demonstrated a slow release rate in both PBS and simulated synovial fluid, with the cumulative release amount reaching up to 80% of the total drug load (Fig. 2F). Calculation of the drug loading confirmed 74.4 ± 2.65% SaB content in SaB NAs. The significantly higher drug loading in SaB NAs compared with other polyphenol-based nanocarriers [21,[30], [31], [32], [33], [34], [35], [36]] (Fig. 2G) demonstrates the ultra-high drug loading capacity of SaB NAs, attributed to their pure drug nano-assemblies. Additionally, TPP serves as a mitochondrial targeting vector and also functions as a therapeutic agent. Free SaB effectively eliminate ROS produced by chondrocytes (Fig. S3), and DPPH assay was used to detect the antioxidant activity of SaB NAs (Fig. S7). Upon co-incubation with SaB NAs at varying concentrations, the DPPH solution progressively faded from purple to pale yellow, with the characteristic absorption peak at 517 nm diminishing nearly completely. At 30 μg/mL, SaB NAs achieved over 90% free radical scavenging capacity (Fig. 2H). SaB NAs retained the superior antioxidant efficacy of free SaB, which is beneficial for the subsequent inhibition of OA.

### 3.3 Self-assembly mechanism of SaB NAs

**Fig. 3:**
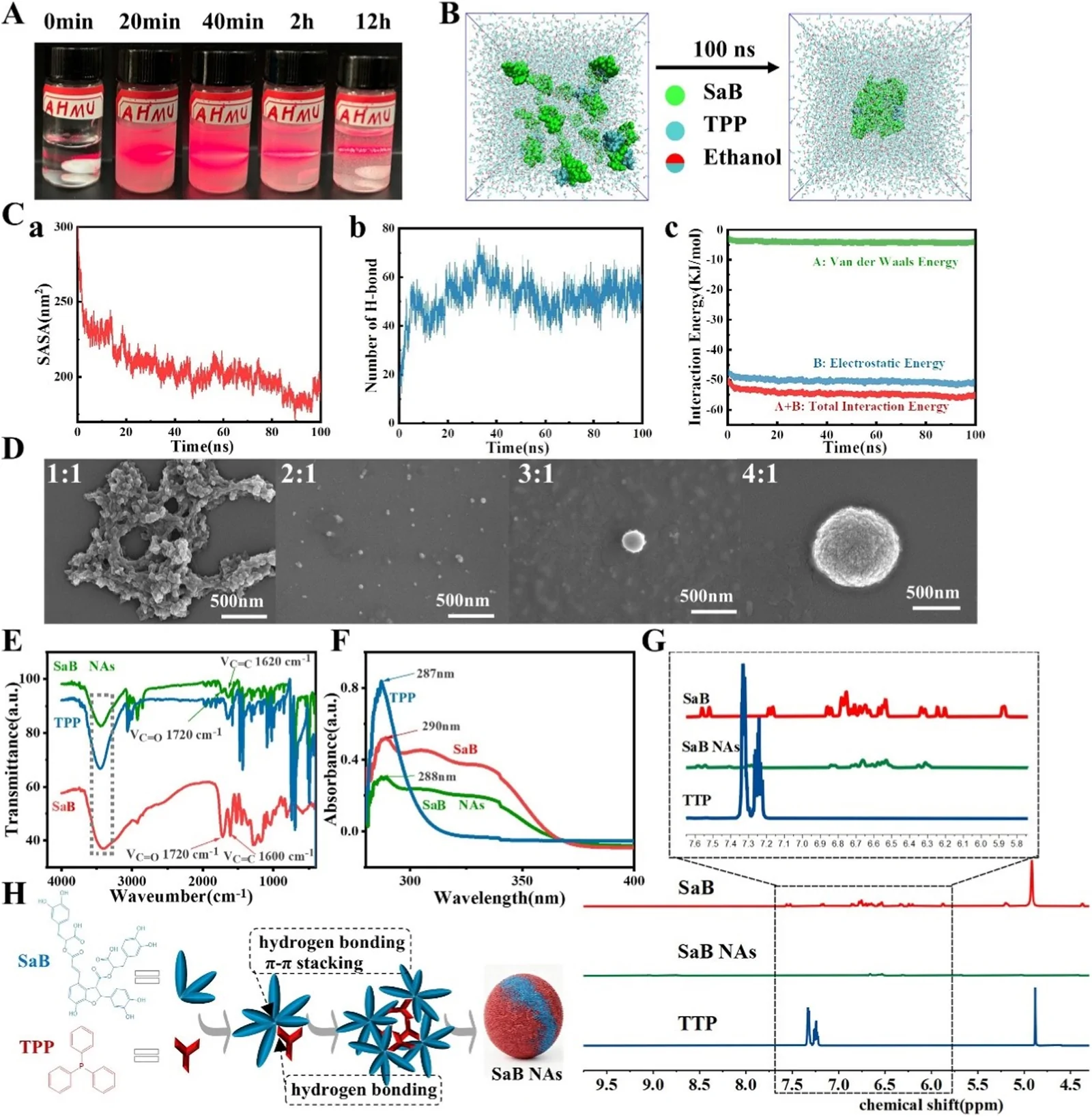
Self-assembly mechanism of SaB NAs. A. State changes of resuspended SaB NAs under red light irradiation over reaction time. B. Structural changes between initial and final states in SaB NAs system simulation. C. Time evolution of a) SASA, b) number of hydrogen bond and c) interaction energy of SaB NAs. D. SEM images of SaB:TPP mixtures at different molar ratios. E. FT-IR spectra of SaB, TPP, and SaB NAs. F. UV-vis spectra of SaB, TPP, and SaB NAs. G. 1H NMR spectra of SaB, TPP, and SaB NAs. H. Self-assembly process diagram of SaB NAs.

To explore the self-assembly process, SaB NAs synthesis was analysis based on different solvent, time and mol ratio. The TPP was dissolved in solvent of varying polarity (H_2_O, methanol, ethanol, acetone and DCM), followed by addition to an aqueous SaB solution. The methanol and ethanol-based TPP solution yielded colloidal suspensions (Fig. S8). Hydrophobic domains within SaB molecules drive hydrophobic interactions, promoting self-assembly under appropriate polar solvents (methanol, ethanol). For safety considerations, ethanol was chosen for subsequent experiments. As the reaction proceeds, colloidal dispersions of SaB NAs were gradually formed. As illustrated in Fig. 3A, the Tyndall effect was observed in the nanoparticle dispersion after 2h of reaction between SaB and TPP. The molecular dynamics simulations revealed a spontaneous aggregation of the SaB NAs system into a nano-cluster structure of the at a 100 ns simulation period (Fig. 3B). Fig. S9A presents molecular dynamics snapshots of the SaB NAs, with conformations captured every 20 ns. Initially, SaB and TPP were randomly distributed in the continuous phase. Subsequently, the SaB NAs became tightly interlinked, forming a spherical structure within the 100 ns simulation timeframe. The molecular assembly process into nanostructures was characterized by a progressive reduction in solvent accessible surface area (SASA) [37]. A significant decrease in SASA was observed in Fig. 3Ca, further indicating the formation of nanostructure. During the entire simulation of the system, the average value of hydrogen bond was 51.458 (Fig. 3Cb). A higher hydrogen bond count indicates that SaB, with its multiple hydroxyl groups, enables extensive hydrogen bonding to form stable, well-organized nanostructures. Additionally, the presence of rigid aromatic backbone within SaB molecules can drive π-π stacking interactions. The interaction energies are presented in Fig. 3Cc. The average electrostatic energy (encompassing hydrogen and ionic bonds) and van der Waals energy (driving π-π stacking) were −50.506 kJ/mol and −4.036 kJ/mol, respectively, indicating that hydrogen bonding is the primary driving force for nanoparticle self-assembly. The molecular interaction patterns in Fig. S9 demonstrated that SaB molecules drive self-assembly through intermolecular and intramolecular hydrogen bonding, as well as π-π stacking between aromatic rings. SaB also forms intermolecular anion-π interactions, which are facilitated by hydrogen bonds between SaB and TPP molecules. The molar ratio between SaB and TPP plays a crucial role in the self-assembly of SaB NAs. As shown in Fig. 3D, no nano spheres were observed at a 1:1 M ratio, indicating that SaB could not provide sufficient bonding to enable self-assembly. As SaB content increased, SaB NAs formed and grew in size. Based on the optimal size requirements for OA treatment discussed as discussed in section 3.2, a 3:1 M ratio was selected for subsequent experiments.

FT-IR was **applied** to reveal changes in the molecular vibrations of the constituent compounds (Fig. 3E). A broad and intense peak covering the range of 3200-3500 cm^-1^ was observed, assigned to the O-H stretching vibration, which facilitate the hydrogen bonding formation. A distinct peak at 1720 cm^-1^ in SaB and SaB NAs, attributed to C=O stretching of the carbonyl groups. This peak retained its position indicating no covalent reaction but rather non-covalent hydrogen bonding. The aromatic C=C stretching vibration at 1600 cm^-1^ of SaB blue-shifted to 1620 cm^-1^ in SaB NAs, consistent with π-π stacking interactions. The UV-Vis absorption spectrum was shown in Fig. 3F. The UV-Vis absorption peaks of SaB and TPP occurred at 287 nm and 290 nm, respectively. The UV-Vis absorption maximum of SaB NAs was at 288 nm, confirming the simultaneous presence of both SaB and TPP in the nanoassemblies. And the broadened and attenuated peak indicated enhanced intermolecular interactions. To confirm the constitution of the nano-assembly, a 1H NMR was conducted. As shown in Fig. 3G, the distinct peak at δ 7.55 ppm vanished, confirming deprotonation of phenolic hydroxyl groups in SaB during SaB NAs formation. Concurrently, the resonances at δ 7.15 ppm exhibited downfield shifts to 6.65 ppm, respectively, indicating π–π stacking interactions between aromatic moieties. Using appropriate polar solvents, a suitable molar ratio, and 2 h of stirring, SaB and TPP self-assemble into nanospheres by hydrogen bonding and π-π stacking (Fig. 3H). This approach provides a rapid and convenient method for synthesizing SaB nanospheres.

### 3.4 Cellular uptake and mitochondrial targeting

**Fig. 4:**
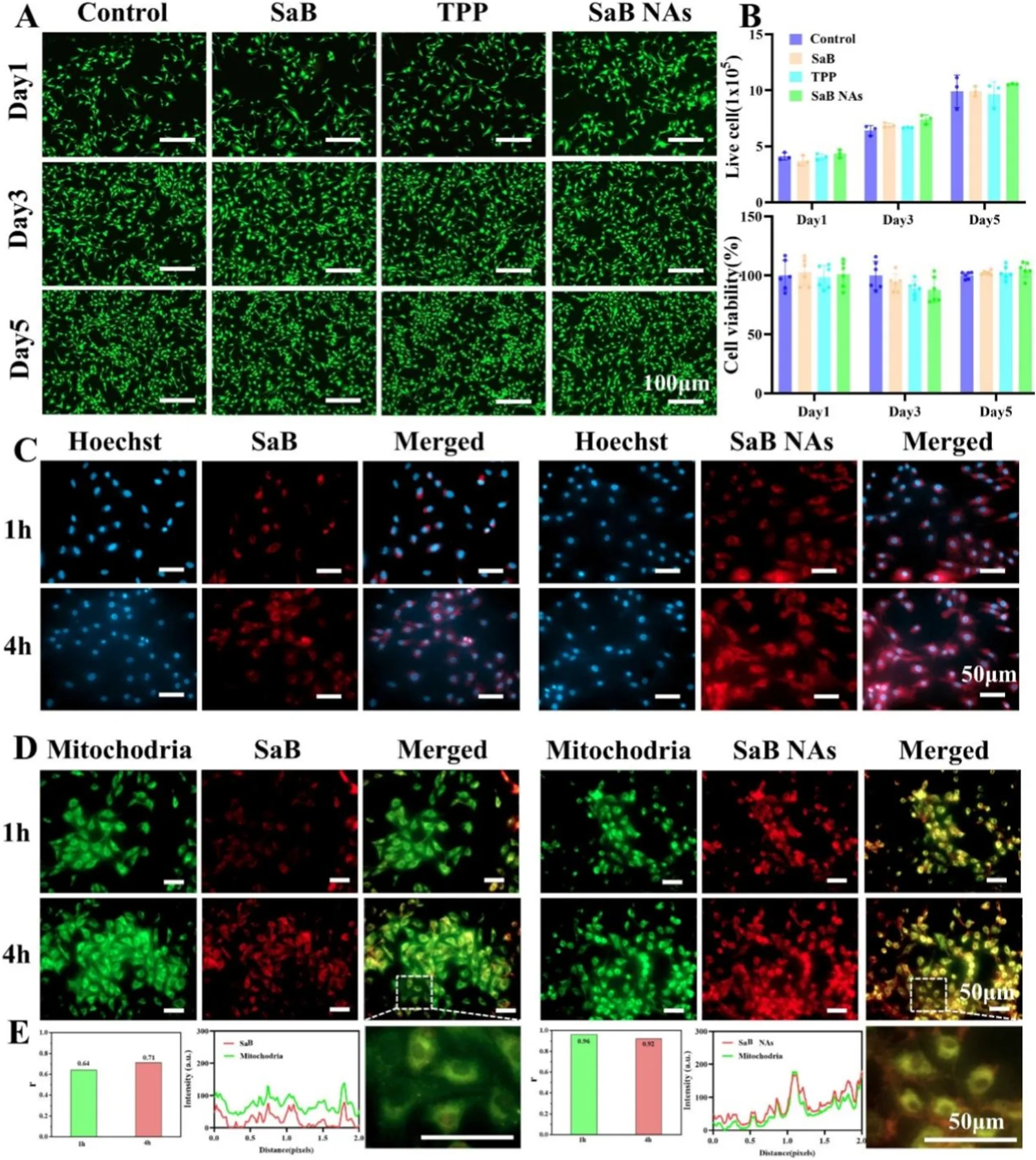
The analysis of cell biocompatibility, cellular uptake and mitochondrial targeting in chondrocyte. A. Fluorescence microscopy images for live/dead staining of cells co-cultured with the materials (scale bar, 100 μm). B. Quantification evaluation of cell number and CCK-8 assay results at 1, 3, and 5 days. C. The confocal images of the SaB and SaB NAs colocalized with chondrocytes for 1 and 4 h, respectively (scale bar, 50 μm). D. The confocal images of the SaB and SaB NAs colocalized with mitochondria for 1 and 4 h, respectively. Red: SaB and SaB NAs with Cy5; Green: Mitochondria stained with Mito-tracker green (scale bar, 50 μm). E. Pearson correlation analysis and line-scan intensity profiles of SaB/SaB NAs and mitochodria signals.

Chondrocytes were incubated with free SaB, TPP, or SaB NAs for 1, 3, or 5 days, followed by cell viability staining. As shown in Fig. 4A, over 90% of chondrocytes remained viable (green staining) after 5 days of treatment, indicating excellent biocompatibility of the tested samples. Cell counting and CCK-8 assays further confirmed that the free-SaB, TPP, and SaB-NAs groups exhibited no obvious cytotoxicity over the 5-day culture period (Fig. 4B). Cy5-labeled free SaB and SaB NAs were utilized to observe the uptake ability of chondrocytes. As illustrated in Fig. 4C, following a 1-h incubation period in an equivalent dose, cells exposed to SaB NAs demonstrated markedly enhanced fluorescence intensity with distinct cytoplasmic localization, in contrast to the free SaB. This result indicates rapid endocytosis of SaB NAs within the initial hour. When incubation duration was extended to 4 h at identical concentrations, a remarkable increase in intracellular fluorescence intensity was observed for SaB NAs. This indicates that SaB NAs as the nano-assemblies can significantly improve the cellular uptake of SaB.

The mitochondrial targeting efficacy of SaB NAs in chondrocytes was assessed using MitoTracker Green as a reference probe (Fig. 4D and E). Pearson correlation analysis and the line-scan intensity profiles revealed that at 1 h, free SaB exhibited negligible red fluorescence intensity with no discernible cytoplasmic accumulation, whereas SaB NAs demonstrated significantly enhanced red fluorescence with pronounced co-localization to mitochondria (yellow signal). At 4 h, the free SaB group showed only a modest elevation in red fluorescence intensity relative to 1 h, but maintained low signal intensity without obvious mitochondrial co-localization. Conversely, the SaB NAs group sustained high red fluorescence intensity with stable yellow co-localization signal, confirming specific mitochondrial targeting capability. SaB NAs demonstrated significantly enhanced cellular uptake kinetics and efficient translocation across cellular membranes compared with free SaB, enabling precise mitochondrial targeting within chondrocytes. This targeted delivery mechanism is primarily mediated by the TPP ligand surface-modified on the nanoparticles. The lipophilic triphenyl moiety of TPP facilitates strong membrane interactions through hydrophobic forces, thereby promoting efficient endocytosis. And the positively charged phosphorus atoms at the molecular termini exploit the mitochondrial membrane potential gradient to drive specific accumulation of nanoparticles within the mitochondrial matrix. This dual-action mechanism establishes the foundation for that SaB NAs preferably regulate chondrocyte function via the mitochondrial autophagy pathway.

### 3.5 Mitochondrial and chondrocyte functions

**Fig. 5:**
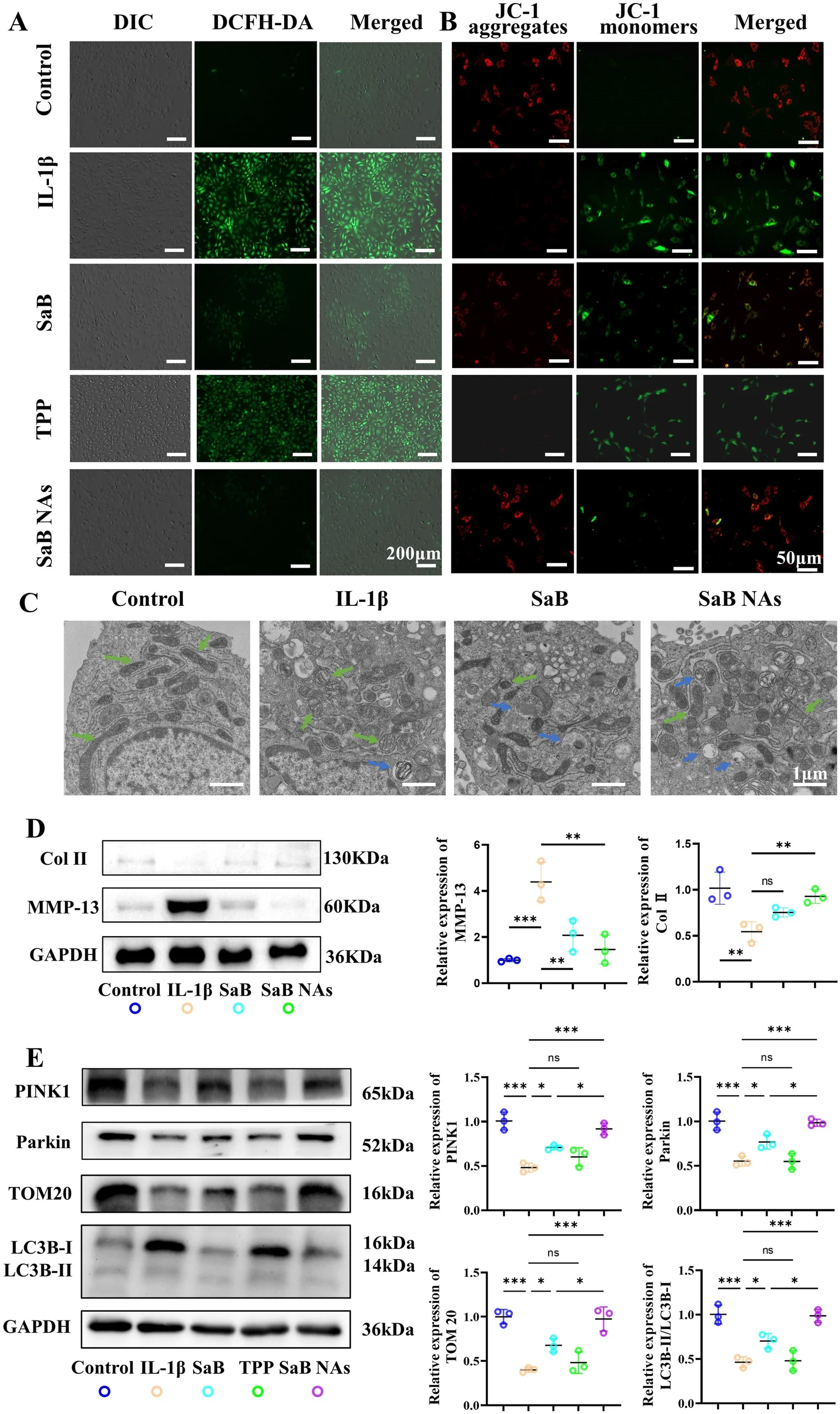
The analysis of mitochondrial and chondrocyte functions. A. Fluorescent images of ROS in IL-1β-stimulated chondrocytes treated with SaB, TPP and SaB NAs (scale bar, 200 μm). B. Representative JC-1 fluorescence images showing mitochondrial membrane depolarization in IL-1β-stimulated chondrocytes treated with SaB, TPP and SaB NAs (scale bar, 50 μm). C. The TEM images of the mitochondria in IL-1β-stimulated chondrocytes treated with SaB and SaB NAs. (scale bar, 1 μm). D. Chondrocyte viability after stimulation in IL-1β-stimulated chondrocytes treated with SaB and SaB NAs. E. The protein expression levels of PINK1, Parkin, TOM20, LC3B-Ⅰ, LC3B-Ⅱ in IL-1β-stimulated chondrocytes treated with SaB, TPP, SaB NAs and quantification analysis. The values are presented as the means ± SDs. *p < 0.05, **p < 0.01, ***p < 0.001, ns, not significant. One-way ANOVA was used for anaysis.

Intracellular ROS levels were measured using the DCFH-DA fluorescent probe. As shown in Fig. 5A, IL-1β treatment significantly increased fluorescence intensity compared with the control group, indicating elevated ROS levels. TPP treatment could not sufficiently reduce the fluorescence intensity elevated by IL-1β stimulation. The SaB NAs group exhibited significantly lower fluorescence intensity than the free SaB group, demonstrating that SaB NAs effectively scavenged intracellular ROS, thereby promoting a regenerative microenvironment for chondrocyte repair. The mitochondrial membrane potential (ΔΨm) of chondrocytes was assessed via JC-1 staining. IL-1β treatment induced a significant reduction in ΔΨm compared with the control group, indicating mitochondrial dysfunction. Following treatment with free SaB or SaB NAs, both groups exhibited markedly increased red fluorescence intensity (JC-1 aggregates) and decreased green fluorescence intensity (JC-1 monomers) relative to the IL-1β group. Notably, at equivalent doses, SaB NAs demonstrated significantly higher red fluorescence intensity in mitochondrial aggregates than free SaB (Fig. 5B). These results indicate that both free SaB and SaB NAs effectively counteract IL-1β-induced ΔΨm depolarization, with SaB NAs exhibiting superior efficacy due to TPP-mediated mitochondrial targeting. TEM was employed to visualize ultrastructural changes in mitophagy-related organelles following treatment (Fig. 5C). The control group exhibited mitochondria with intact morphology, well-defined cristae, and regular arrangement. In contrast, IL-1β-treated chondrocytes displayed severe mitochondrial damage, including swelling, cristae disruption, and vacuolation, indicating impaired mitophagy that failed to clear damaged organelles, thereby exacerbating cellular injury. Free SaB treatment partially restored mitochondrial structure with improved cristae density, suggesting enhanced mitophagy. Critically, SaB NAs treatment restored near-normal mitochondrial morphology, with well-defined cristae and markedly diminished swelling/vacuolation. Based on this, SaB NAs can more effectively restore chondrocyte function than free SaB (Fig. 5D). SaB NAs increased the expression level of Col II, and simultaneously decreased expression level of MMP-13, suggesting the SaB NAs could facilitate cell matrix synthesis and inhibit the expression of related matrix-degrading enzymes. To further elucidate the molecular mechanisms by which SaB triggers mitophagy in chondrocytes, we detected the protein expression of core regulators in the PINK1/Parkin mitophagy pathway (Fig. 5E). For the IL-1β group, the expression of PINK1 and Parkin was significantly downregulated. Following treatment with different groups, single TPP treatment exerted no obvious regulatory effect on these mitophagy-related proteins, while the SaB and SaB NAs groups displayed elevated expression. Notably, the SaB NAs group demonstrate a more pronounced effect compared to the SaB group. Furthermore, the IL-1β-treated group exhibited a significant downregulation of the mitochondrial marker TOM20, accompanied by a marked decrease in the conversion of LC3B-II/LC3B-I ratio and compared to the control group. Treatment with SaB or SaB NAs effectively reversed these pathological changes. Notably, the SaB NAs formulation demonstrated superior efficacy compared to free SaB, leading to more robust stabilization of PINK1/Parkin and a higher LC3B-II/LC3B-I ratio. Collectively, these results suggest that SaB and SaB NAs may activate PINK1/Parkin-dependent mitophagy, thereby facilitating the selective elimination of dysfunctional mitochondria in chondrocytes.

### 3.6 RNA-sequencing

**Fig. 6:**
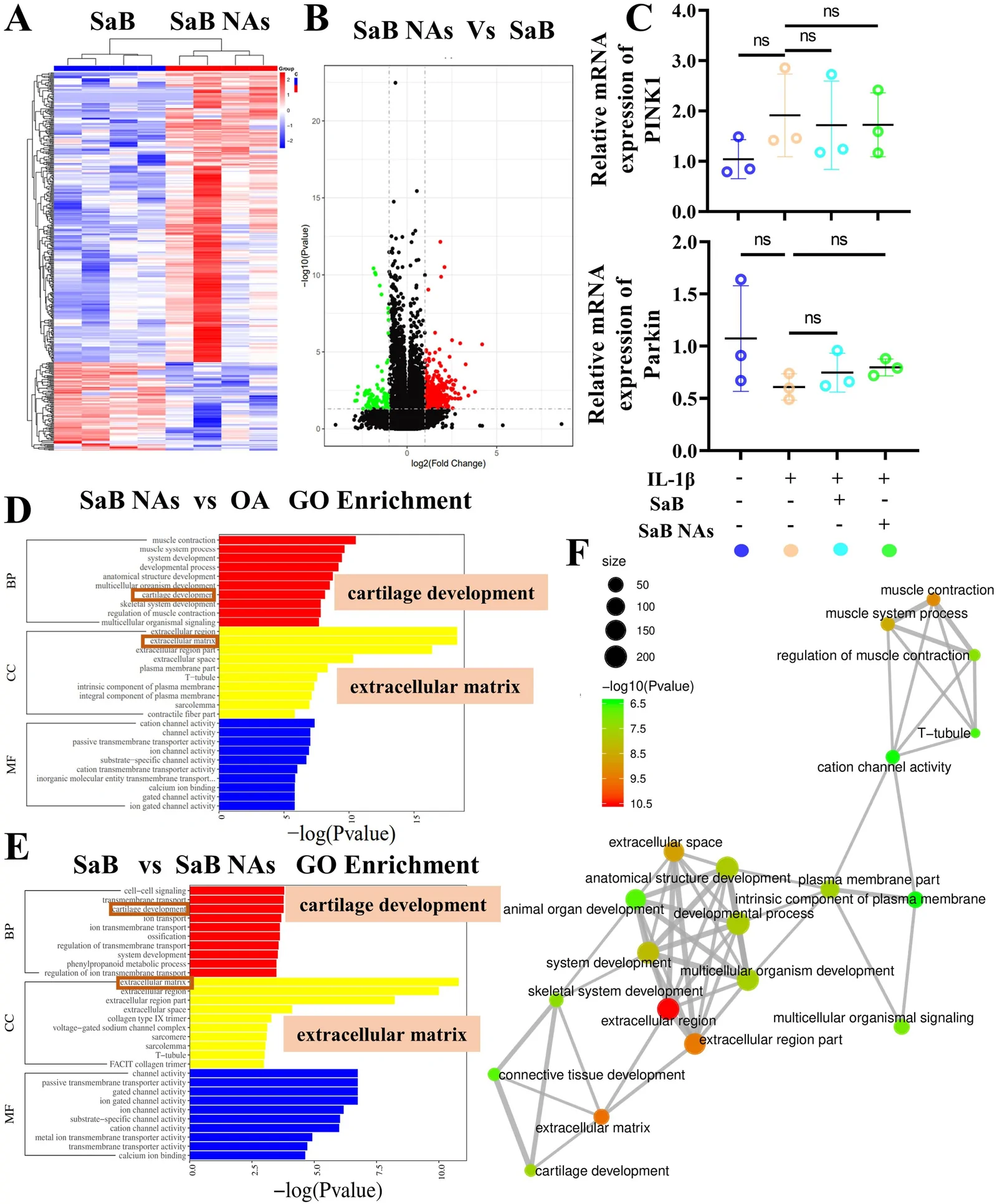
The analysis of therapeutic effect by transcriptomic analysis. A. Heatmap and B. Volcano plot of differentially expressed genes between SaB and SaB NAs. C. qRT-PCR results of PINK1 and Parkin expressions for chondrocytes (ns, not significant). Enrichments in GO pathways separately shown in biological process (BP), cellular component (CC), and molecular function (MF) subgroups for D. SaB NAs and OA. E. SaB and SaB NAs. F. GO enrichment analysis network diagram. ns, not significant. One-way ANOVA was used for anaysis.

To elucidate the underlying mechanism of SaB and SaB NAs in facilitating mitochondrial and chondrocyte function, the mRNA sequencing between SaB, SaB NAs and OA group was compared with analyze the differential gene expression of OA model in treatment with SaB and SaB NAs, respectively. As shown in Fig. 6A and Fig. S10A, the differentially expressed genes (DEGs) analysis revealed that biological replicates within each group clustered tightly together, meeting the requirements for subsequent functional enrichment analysis. In Fig. S10B, volcano plot analysis revealed 193 DEGs in the SaB group vs OA (116 up-regulated, 77 down-regulated), while the SaB NAs group exhibited 1029 DEGs (620 up-regulated, 409 down-regulated). Comparative analysis between SaB and SaB NAs groups identified 441 DEGs (Fig. 6B), demonstrating that SaB NAs exerts a significantly more potent regulatory effect on OA chondrocytes than free SaB. Interestingly, DEGs analysis revealed no significant difference in PINK1 and Parkin mRNA expression levels, consistent with the qRT-PCR results of PINK1 and Parkin expressions for chondrocytes (Fig. 6C). While GO enrichment analysis demonstrated that SaB NAs group were significantly enriched in cartilage development and extracellular matrix pathways (Fig. 6D), with stronger enrichment compared with the SaB group (Fig. 6E), indicating SaB NAs more effectively activates molecular mechanisms driving cartilage repair in OA chondrocytes. From Fig. 6F, the significant enrichment of DEGs indicate that the SaB NAs confers chondroprotective effects by maintaining cartilage extracellular matrix homeostasis and promoting chondrocyte differentiation.

The above results in PINK1 and Parkin gene transcription remained unchanged while PINK1 and Parkin protein levels increased significantly, reveals that SaB and SaB NAs binding to PINK1 and Parkin protein enhancing protein stability and promoting its expression. This cascade activates mitophagy, upregulates extracellular matrix synthesis, and accelerates cartilage development. Due to the advantages of SaB NAs in terms of nanoscale structures and targeting mitochondria, SaB NAs exhibit a better therapeutic effect.

**Fig. 7:**
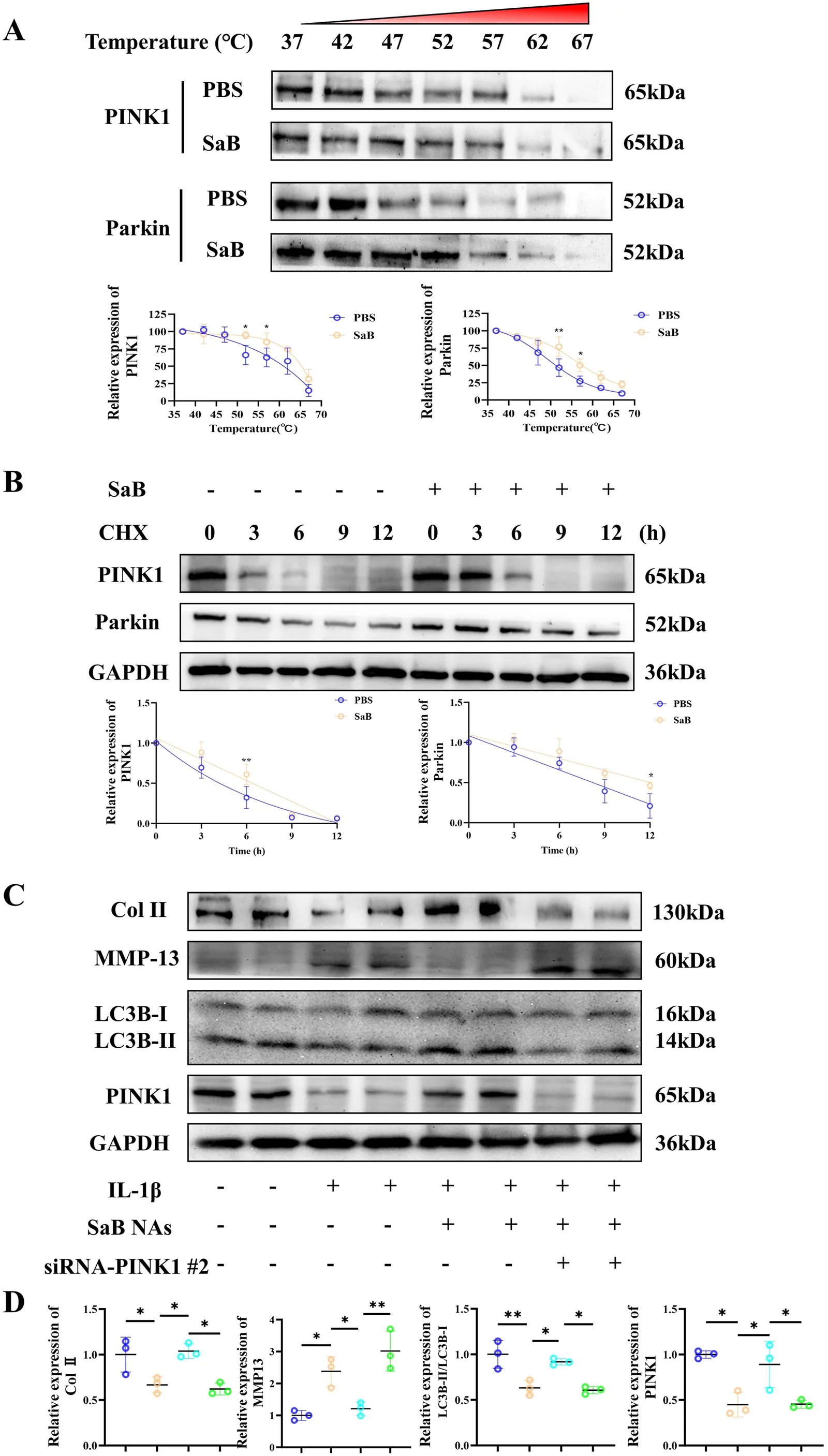
Integrated analysis of thermal stability, degradation kinetics, and signaling modulation. A. The CETSA of PINK1 and Parkin thermal stability in chondrocytes treated with PBS or SaB. B. The CHX chase assay of PINK1 and Parkin protein degradation kinetics in chondrocytes treated with PBS or SaB. Two-way ANOVA was used for anaysis. Two-way ANOVA was applied to analyze. C. Western blot analysis of Col II, MMP-13, LC3B II/LC3B-I and PINK1 in chondrocytes treated with or without IL-1β and SaB NAs, following transfection with control siRNA or PINK1-specific siRNA #2. D. Quantitative analysis of relative protein expression for Col II, MMP-13, LC3B, and PINK1. *p < 0.05, **p < 0.01, One-way ANOVA was used for anaysis.

To investigate whether SaB directly engages PINK1/Parkin in the cellular context, the CETSA was employed. As shown in Fig. 7A, the PINK1 and Parkin in PBS-treated cells exhibited a massive thermal denaturation, with substantial loss of soluble protein occurring between 47°C and 62°C. Strikingly, pre-treatment with SaB resulted in a pronounced rightward shift in the melting curves of both proteins, indicating that SaB significantly enhanced the thermal stability. These CETSA data suggest that SaB may stabilize cellular PINK1 and Parkin mainly through direct binding. Furthermore, a CHX chase assay was performed as depicted in Fig. 7B. In PBS-treated control cells, PINK1 and Parkin protein levels declined progressively over the 12 h CHX treatment period. Notably, pre-treatment with SaB markedly attenuated the degradation of both proteins. These results align well with the CETSA findings and reinforces the notion that SaB binds to PINK1/Parkin proteins to stabilize them. To ascertain whether the chondroprotective effects of SaB NAs are specifically mediated through PINK1, we silenced PINK1 expression using siRNA under in vitro drug treatment conditions. Efficient knockdown of PINK1 was achieved in chondrocytes with siRNA-PINK1 #2 (Fig. S11). As shown in Fig. 7C and D, IL-1β stimulation markedly downregulated Col II and strongly upregulated MMP-13, recapitulating the key features of inflammatory cartilage degradation. These pathological alterations were substantially ameliorated by co-treatment with SaB NAs. However, such protective effects were nearly completely abrogated following siRNA-mediated PINK1 knockdown. These findings suggest that the anti-catabolic and pro-anabolic activities of SaB NAs in chondrocytes are strictly dependent on PINK1 expression. In parallel, we assessed how PINK1 knockdown affects SaB NAs-triggered autophagy by determining the LC3B-II/LC3B-I ratio. IL-1β exposure suppressed basal autophagic activity, whereas co-treatment with SaB NAs significantly restored autophagy toward control levels. Importantly, this restorative effect was largely abolished upon PINK1 silencing, indicating that SaB NAs promote autophagy in IL-1β-stimulated chondrocytes via a PINK1-dependent signaling axis.

### 3.7 In vivo OA amelioration

**Fig. 8:**
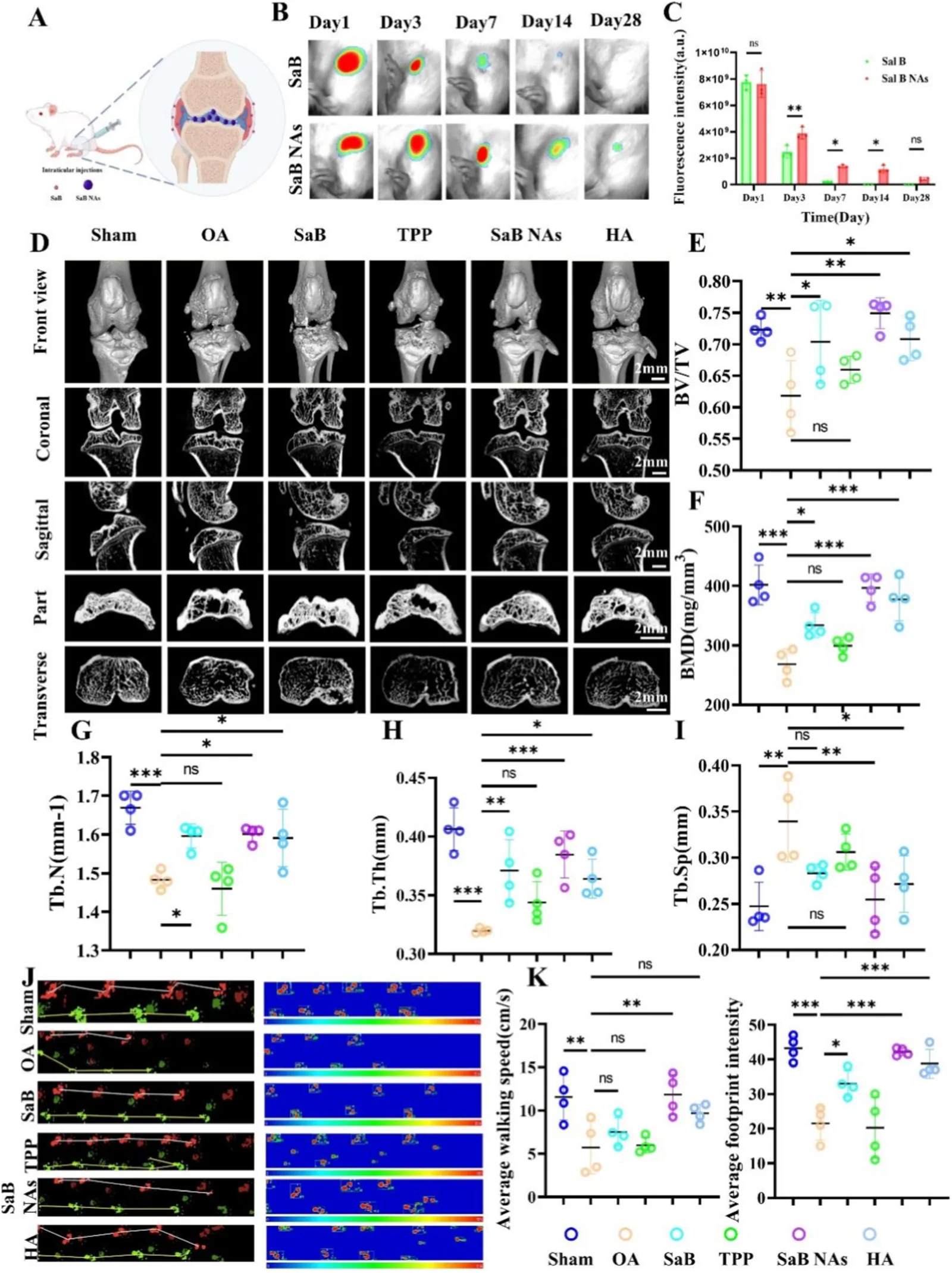
*In vivo* retention and therapeutic efficacy. A. The release of SaB and SaB NAs in in the intra-articular cavity of OA model rats. B. Representative IVIS images of rat knee joints within a four-week period. C. Quantitative analysis of the time-course fluorescent radiant efficiency. *t*-test was applied. D. Micro-CT with 3D reconstruction and representative coronal, sagittal, part and transverse CT images of the rat knee joint (scale bar, 2 mm). The effect of treatment on the values of E. bone volume ratio (BV/TV), F. Bone mineral density (BMD), G. Trabecular number (Tb.N), H. Trabecular thickness (Tb.Th) and I. trabecular separation (Tb.Sp). J. Representative images of gait and K. Quantitative analysis of walking speed and footprint intensity. The values are presented as the means ± SDs. *p < 0.05, **p < 0.01, ***p < 0.001, ns, not significant. One-way ANOVA was used for anaysis.

As previously analyzed, the SaB NAs (340 nm) can delay rapid clearance from the joint cavity. To evaluate the joint cavity retention characteristics of SaB NAs in rats, an *in vivo* fluorescence tracing study was conducted using a small animal live imaging system (Fig. 8A). As shown in Fig. 8B and C, fluorescence intensity in the joint of the free SaB group declined rapidly, becoming undetectable by day 7 post-administration. In contrast, the SaB NAs group maintained a strong fluorescence signal in the joint area up to day 14. These results demonstrate that SaB NAs exhibit significantly superior retention capability and prolonged residence time in the joint cavity compared with free SaB. This sustained retention profile provides a critical foundation for the continuous and stable chondrocyte reparative activity of SaB NAs within the joint microenvironment. To evaluate the *in vivo* therapeutic efficacy, a series of drug treatments was conducted in OA model rats. Among them, hyaluronic acid (HA), a clinically established treatment for OA, was administered as the reference therapeutic agent in this study. Five weeks post-therapeutic intervention, rat knee joints were subjected to Micro-CT scanning and three-dimensional reconstruction. Micro-CT analysis (Fig. 8D) revealed intact cartilage surfaces in the sham-operated group, while the OA model group exhibited severe cartilage degeneration on the femoral condyles and tibial plateaus, characterized by surface roughness, osteophyte formation, and impaired joint function. SaB NAs treatment group significantly reduced osteophyte formation and promoted partial cartilage surface repair. Sagittal, coronal, and cross-sectional reconstructions demonstrated that OA model rats displayed disrupted trabecular architecture in the femur and tibia, with sparse trabecular networks and peripheral osteophyte formation. Quantitative bone analysis (Fig. 8E–I) confirmed that OA rats exhibited significantly decreased trabecular parameters compared with the control group. While free SaB and TPP groups showed minimal improvement in these parameters, the SaB NAs group demonstrated significant improvement of the bone parameters and trabecular structure, confirming its efficacy in inhibiting osteophyte formation and promoting cartilage-protective effects. The excellent therapeutic effects of SaB NAs were further confirmed in histological images of the knee joint at 5 weeks postoperatively (Fig. S12). Gait analysis was performed using a dynamic force plate system to assess motor function recovery and pain-related behavioral changes in all rat groups (Fig. 8J–K). Sham-operated rats exhibited normal gait patterns with coordinated limb movement and balanced weight-bearing distribution. In contrast, OA model rats demonstrated significant gait abnormalities, characterized by reduced average walking speed and markedly diminished footprint pressure. SaB NAs group significantly restored walking speed and footprint pressure, indicating effective alleviation of gait abnormalities and joint pain.

**Fig. 9:**
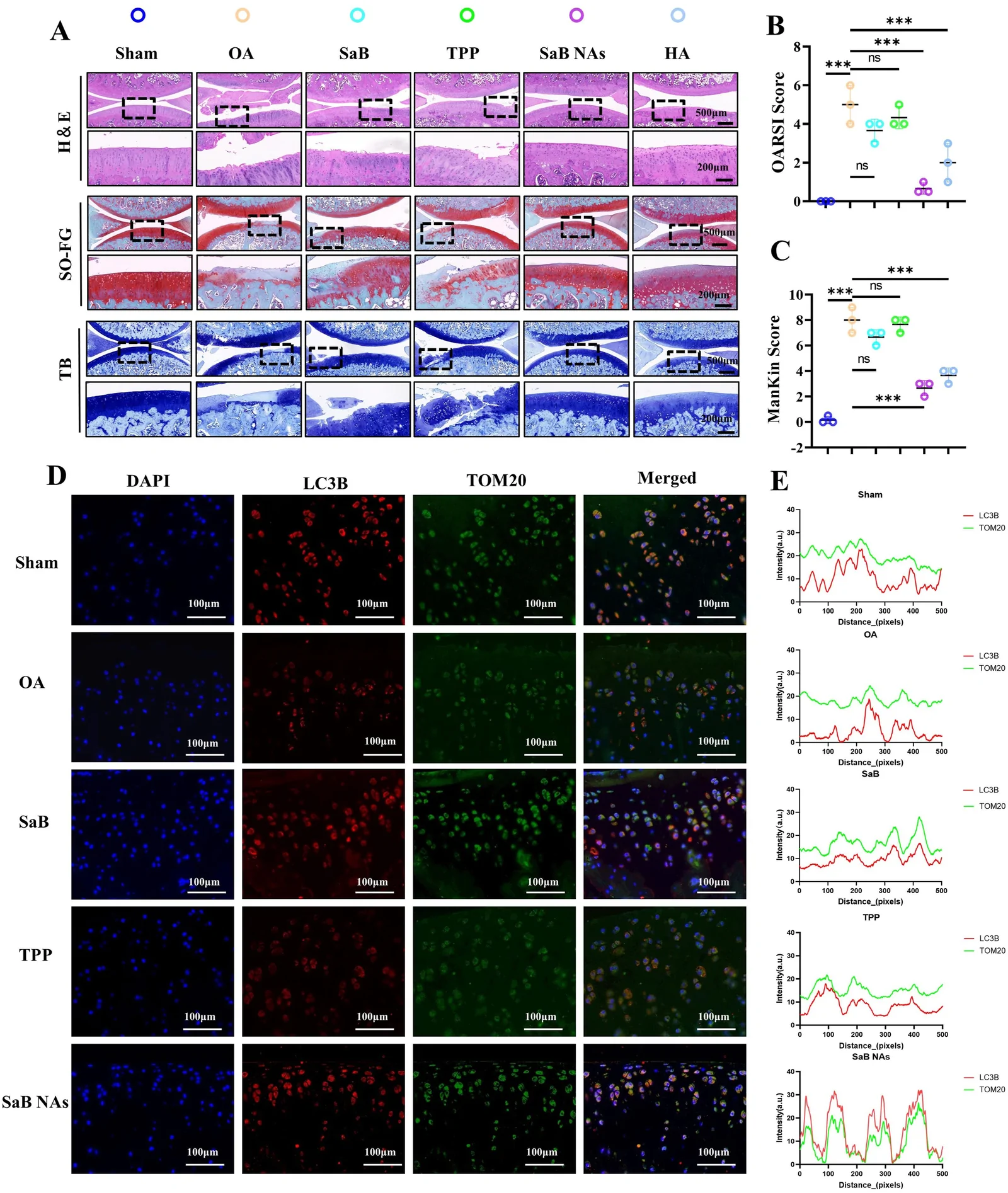
Histological evaluation of the therapeutic effect. A. Representative morphology of the rat knee joint was detected by H&E, SO-FG and TB staining (scale bar for top images, 500 nm, scale bar for bottom images, 200 nm). B. Osteoarthritis Research Society International (OARSI) and C. Markin score at 5 weeks postoperatively. D. Representative immunofluorescence images of cartilage sections from Sham, OA, SaB, TPP, and SaB NAs groups stained for LC3B (red), TOM20 (green), and nuclei (DAPI, blue) (scale bar, 100 μm). E. Line-scan intensity profiles of LC3B and TOM20 signals. The values are presented as the means ± SDs. *p < 0.05, **p < 0.01, ***p < 0.001, ns, not significant. One-way ANOVA was used for anaysis.

H&E, safranin O-fast green and toluidine blue staining were used to compare histological changes on the cartilage surfaces in different treatment groups (Fig. 9A). Compared with the sham group, typical morphological changes, including severe cartilage erosion and significant thinning was observed in OA group. Treatment with SaB and TPP exhibited no reduction in cartilage damage, whereas the SaB NAs group revealed superior efficacy in preserving cartilage architecture and minimizing degenerative changes. The OARSI (Fig. 9B) and Mankin (Fig. 9C) scores demonstrated that the SaB NAs group exhibited superior efficacy in attenuating osteoarthritic degeneration compared with all other experimental groups. Immunofluorescence staining was performed to detect LC3B and TOM20, a specific marker protein of the mitochondrial outer membrane, in cartilage tissues from different treatment groups. The colocalization of LC3B and TOM20 indicates the occurrence of mitophagy. As shown in Fig. 9D, the sham group exhibited a baseline level of LC3B puncta with evident colocalization with TOM20, reflecting normal physiological mitophagy. In contrast, the OA group showed significantly reduced LC3B fluorescence intensity and decreased colocalization between LC3B and TOM20. These findings suggest that mitophagy flux is impaired under OA pathological conditions, which may lead to the accumulation of dysfunctional mitochondria and subsequent chondrocyte stress. Compared with the OA group, the SaB-treated group exhibited markedly increased LC3B puncta formation and restored LC3B-TOM20 colocalization, indicating that SaB could reactivate mitophagy and facilitate the clearance of damaged mitochondria. Notably, merged immunofluorescence images and corresponding fluorescence intensity profiles (Fig. 9E) revealed substantially enhanced colocalization of LC3B and TOM20 in the SaB NAs group. These results suggest that SaB NAs exert superior therapeutic efficacy, which may be attributed to their improved bioavailability and mitochondrial-targeted delivery capability.

**Fig. 10:**
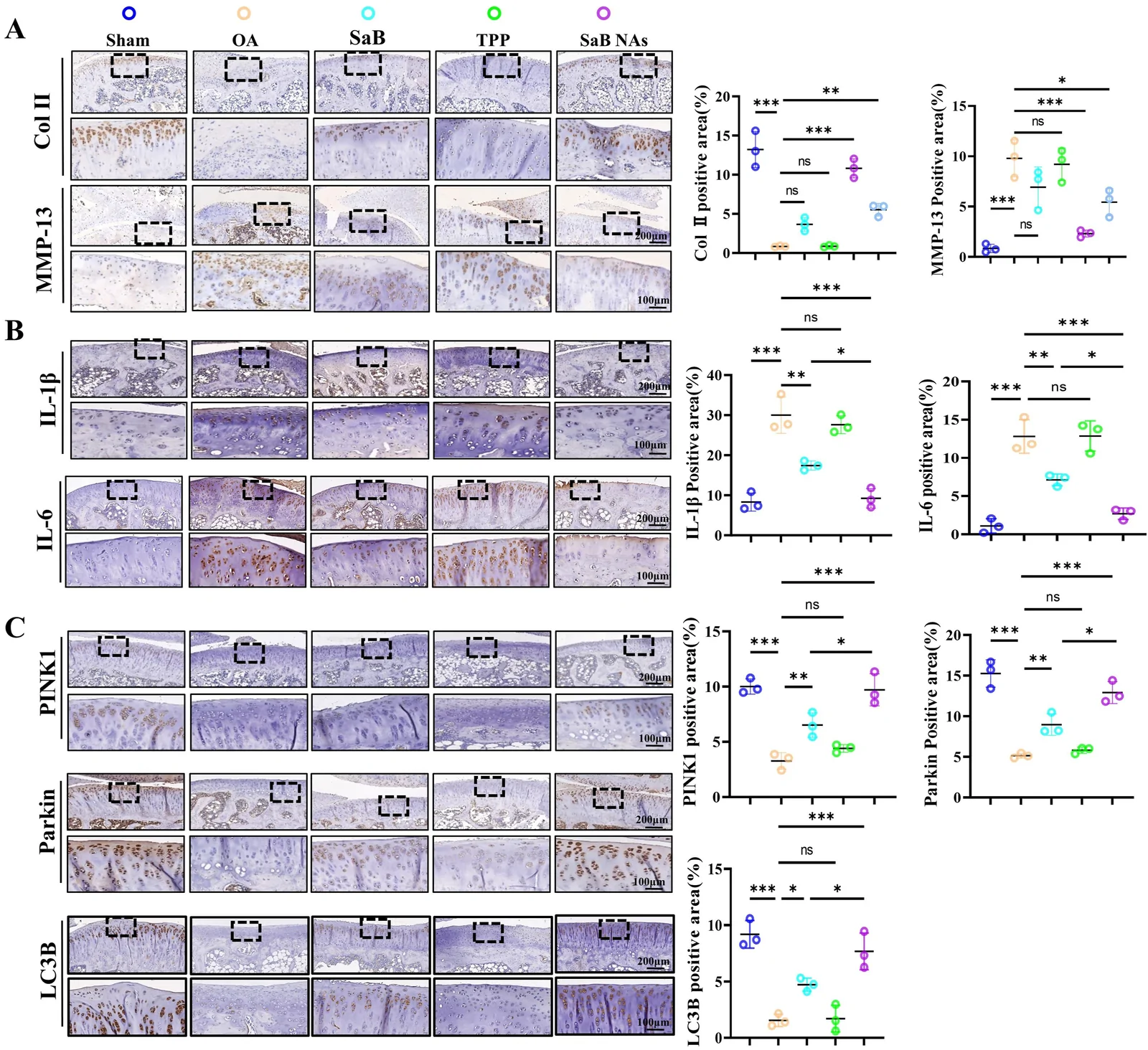
Immunohistochemical analyses in cartilage tissues. Immunohistochemical sections and quantitative analysis of A. Col II and MMP-13 expression. B. IL-1β and IL-6 expression. C. PINK1, Parkin and LC3B expression. (scale bar for top images, 200 nm, scale bar for bottom images, 100 nm). The values are presented as the means ± SDs. *p < 0.05, **p < 0.01, ***p < 0.001, ns, not significant. One-way ANOVA was used for anaysis.

To evaluate whether SaB NAs exert therapeutic effects against cartilage degradation by promoting mitophagy, immunohistochemical staining was performed to assess key markers associated with extracellular matrix metabolism, inflammation, and mitophagy. As shown in Fig. 10, the OA group exhibited a significant downregulation of Col II, accompanied by a marked upregulation of the catabolic enzyme MMP-13, as well as pro-inflammatory cytokines IL-1β and IL-6, indicating severe cartilage destruction and inflammatory response. Conversely, treatment with SaB NAs effectively reversed these pathological changes, restoring Col II expression while significantly suppressing the levels of MMP-13, IL-1β and IL-6, demonstrating superior anti-inflammatory and chondroprotective effects compared with free SaB or TPP groups. Furthermore, regarding the underlying mechanism, the OA group showed diminished expression of PINK1, Parkin, and LC3B, suggesting impaired mitophagy. Notably, SaB NAs treatment significantly upregulated the expression of these mitophagy-related proteins (Fig. 10C), implying that SaB NAs alleviate osteoarthritis progression by activating the PINK1/Parkin-mediated mitophagy pathway to maintain mitochondrial homeostasis. The histopathological assessment of major organs (heart, liver, spleen, lung, and kidney) via H&E staining revealed no histopathological abnormalities in any treatment group relative to vehicle controls (Fig. S13). These findings confirm that the drug treatments in this system have no potential systemic toxicity. The excellent biosafety further supports the clinical advancement of SaB NAs.

## 4 Conclusions

This study identifies SaB as a small-molecule agent capable of restoring impaired mitophagy in chondrocytes through regulation of the PINK1/Parkin pathway. SaB NAs were fabricated relying on hydrogen bonding and π–π stacking interactions between SaB and TPP, with synthetic parameters including solvent, reaction time and molar ratio optimized to realize high drug loading and enable mitochondrial-targeted combinatorial therapy. Cellular experiments revealed that SaB (25-100 μM) upregulated the expression of PINK1/Parkin proteins in a dose-dependent manner, thereby ameliorating mitochondrial dysfunction and counteracting chondrocyte degeneration under OA conditions. Potential binding of SaB to PINK1/Parkin stabilizes these proteins and facilitates their expression, which further activates mitophagy, promotes ECM synthesis and supports cartilage homeostasis. Compared with free SaB, SaB NAs show improved cellular uptake and membrane translocation, contributing to better recovery of mitochondrial and chondrocyte function. In vivo experiments verified that SaB NAs exerted more favorable effects than free SaB and clinically used HA in relieving OA manifestations and slowing disease progression. Earlier work has examined free SaB or SaB-based nanoparticles for the treatment of other conditions[38], against this background, our study makes several meaningful explorations. We confirm that SaB modulates the PINK1/Parkin pathway in chondrocytes, construct carrier-free SaB/TPP co-assembled nanoformulations with high drug loading, and explore the potential of mitochondrial-targeted SaB nano-assemblies for OA treatment. Collectively, this work demonstrates the potential utility of SaB NAs in facilitating cartilage repair, and may offer a feasible strategy for the development of novel OA therapeutic candidates.

## CRediT authorship contribution statement

**Yulin Jiang:** Writing – original draft, Methodology, Investigation, Data curation, Conceptualization. **Mingzhu Wang:** Writing – review & editing, Writing – original draft, Methodology, Investigation. **Hanshuang Liang:** Methodology, Investigation. **Xuejie Hu:** Methodology, Investigation. **Liuwei Chu:** Methodology, Investigation. **Ranran Yang:** Methodology, Investigation, Data curation. **Kang Wang:** Writing – original draft, Investigation. **Ruhong Fang:** Writing – original draft, Methodology, Investigation, Data curation. **Cailiang Shen:** Writing – original draft, Methodology, Investigation, Data curation. **Xinming Wang:** Conceptualization. **Wei Wei:** Writing – review & editing, Conceptualization. **Qingtong Wang:** Writing – review & editing, Funding acquisition, Conceptualization.

## Declaration of competing interest

The authors declare that they have no known competing financial interests or personal relationships that could have appeared to influence the work reported in this paper.

## Data Availability

Data will be made available on request.
