# Supplementary material for: The mitophagy targeted nano-assemblies with high Salvianolic acid B -loading sufficiently enhances osteoarthritis therapy
Source: Mater Today Bio. 2026 Aug 26;40:103601. doi: 10.1016/j.mtbio.2026.103601 (PMC13563592; doi:10.1016/j.mtbio.2026.103601)
Supplement: Supplementary file 1 [file mmc1.docx]

Supporting Information

**The mitophagy targeted nano-assemblies with high Salvianolic acid B - loading sufficiently enhances osteoarthritis therapy**

Yulin Jiang^a,b#^, Mingzhu Wang^a#^, Hanshuang Liang^a^, Xuejie Hu^a^, Liuwei Chu^a^, RanRan Yang^a^, Kang Wang^c^, Ruhong Fang^a^, Cailiang Shen^b^, Xinming Wang^d*^, Wei Wei^a*^, Qingtong Wang^a*^

*^a^* Institute of Clinical Pharmacology, School of Pharmacy, Anhui Medical University; Key Laboratory of Anti-inflammatory and Immune Medicine, Ministry of Education; Anhui Provincial Engineering Technology Research Center for Anti-inflammatory and Immune Drugs, Hefei 230032, China

*^b^* Department of Orthopedics and Spine Surgery, The First Affiliated Hospital of Anhui Medical University, Hefei 230022, China

*^c^* Department of Immunology, School of Basic Medical Sciences, Center for Big Data and Population Health of IHM, Anhui Medical University, Hefei 230032, China

*^d^* Department of Pharmacy, First Affiliated Hospital of Anhui Medical University, Hefei 230022, China

*Corresponding author

Qingtong Wang, E-mail: qingtongwang@ahmu.edu.cn; Tel.: +86055165161206.

Wei Wei, E-mail: wwei@ahmue.edu.cn; Tel.: +86055165161209.

Xinming Wang, E-mail: wangxinming@ahmu.edu.cn; Tel.: +86055165908530

^#^These authors contributed equally to this work.

**Figure S1.** CCK-8 assays of chondrocyte treated with different concentrations of SaB for 1, 3, 5 and 7 days. The values are presented as the means ± SDs. **p*<0.05, ** *p*<0.05, *** *p*<0.001, ns, not significant.

**Figure S2.** JC-1 fluorescence images showing mitochondrial membrane depolarization in IL-1β-stimulated chondrocytes treated with SaB (0, 25, 50, or 100 μM) (scale bar, 25 μm).

**Figure S3.** Fluorescent images of ROS in IL-1β-stimulated chondrocytes treated with SaB (0, 25, 50, or 100 μM) (scale bar, 100 μm).

**Figure S4.** The TEM image of SaB NAs.

**Figure S5.** HPLC spectra of SaB, TPP and SaB NAs.

**Figure S6.** Zeta potential of SaB NAs in culture medium, serum-containing medium, and simulated synovial fluid for seven days.

**Figure S7.** UV-vis spectra of the DPPH ethanol solution incubated with SaB NAs, the equivalent concentrations from (1)-(6) are 0, 2, 4, 8, 20 and 30 μg/mL, respectively.

**Figure S8.** Dissolution of SaB NAs in solvents of varying polarity.

**Figure S9** A. Snapshots of the assembly process of DPNAs captured between 0 ns and 100 ns. B. The molecular interaction patterns in the SaB NAs system.

**Figure S10.** A. Heatmap of differential gene expression between SaB, SaB NAs and OA. B. Volcano plot of upregulated and downregulated genes between SaB, SaB NAs and OA. Bubble plot of C. GO and D. KEGG enrichment analysis of differentially expressed genes between SaB NAs and OA.

**Figure S11.** Validation of PINK1 knockdown efficiency using specific siRNAs.

**Figure S12.** The histological images of the rat knee joint at 5 weeks postoperatively (scale bar, 1 mm).

**Figure S13.** Representative H&E staining of major organs-including the heart, liver, spleen, lung, and kidney-from rats (scale bar, 200 nm).

**Table S1.** Primers used for quantitative real-time polymerase chain reaction.

| Gene | Primer sequences (5′-3′) |  |
| --- | --- | --- |
|  | Forward | Reverse |
| PINK1 | TGTATGAAGCCACCATGCCC | TGGAGGATCCTGCCGAGATA |
| Parkin  GAPDH | GAACTGTGGCTGTGAGTGGA  CACGACATACTCAGCACCAG | GGTGTTTCCCATGAGGTCGT  TCCAGTATGACTCTACCCACG |
